# Development of a bioprinter-based method for incorporating metabolic competence into high-throughput *in vitro* assays

**DOI:** 10.3389/ftox.2023.1196245

**Published:** 2023-05-04

**Authors:** Kristen Hopperstad, Chad Deisenroth

**Affiliations:** Center for Computational Toxicology and Exposure, Office of Research and Development, U.S. Environmental Protection Agency, Durham, NC, United States

**Keywords:** high-throughput screening, xenobiotic metabolism, new approach methods, bioprinting, endocrine toxicology

## Abstract

The acceptance and use of *in vitro* data for hazard identification, prioritization, and risk evaluation is partly limited by uncertainties associated with xenobiotic metabolism. The lack of biotransformation capabilities of many *in vitro* systems may under- or overestimate the hazard of compounds that are metabolized to more or less active metabolites *in vivo*. One approach to retrofitting existing bioassays with metabolic competence is the lid-based Alginate Immobilization of Metabolic Enzymes (AIME) method, which adds hepatic metabolism to conventional high-throughput screening platforms. Here, limitations of the lid-based AIME method were addressed by incorporating bioprinting, which involved depositing S9-encapsulated microspheres into standard 384-well plates with requisite cofactors for phase I and II hepatic metabolism. Objectives of this study included: 1) compare the lid-based and AIME bioprinting methods by assessing the enzymatic activity of a common cytochrome P450 (CYP) enzyme, 2) use biochemical assays with the bioprinting method to characterize additional measures of phase I and II metabolic activity, and 3) evaluate the bioprinting method by screening 25 chemicals of known metabolism-dependent bioactivity in the VM7Luc estrogen receptor transactivation (ERTA) assay. A comparison of the two methods revealed comparable precision and dynamic range. Activity of additional CYP enzymes and glucuronidation was observed using the AIME bioprinting method. The ERTA experiment identified 19/21 ER-active test chemicals, 14 of which were concordant with expected biotransformation effects (73.7%). Additional refinement of the AIME bioprinting method has the potential to expand high-throughput screening capabilities in a robust, accessible manner to incorporate *in vitro* metabolic competence.

## 1 Introduction

Confidence in the implementation of *in vitro* New Approach Methods (NAMs) requires comprehensive evaluation of potential chemical bioactivity to inform human health protection using next-generation risk assessments (Thomas et al., 2019; EPA, 2021; Carmichael et al., 2022; van der Zalm et al., 2022). While *in vitro* NAMs can evaluate the potential effects of parent chemicals, a frequently cited limitation is the lack of coverage for xenobiotic metabolic processes that occur *in vivo* (Kirkland et al., 2007; Jacobs et al., 2013). The lack of biotransformation capabilities of many *in vitro* systems may under- or overestimate the hazard of compounds that are metabolized to more or less active metabolites *in vivo*, partly limiting the acceptance and use of *in vitro* data for hazard identification, prioritization, and risk evaluation. The incorporation of xenobiotic metabolic competence may benefit high-throughput chemical screening (HTS) by decreasing uncertainty associated with extrapolation of *in vitro* data for estimating potential human health hazards. The U.S. Environmental Protection Agency has acknowledged the lack of coverage for xenobiotic metabolic processes in chemical safety research and established a tiered testing paradigm to address challenges in chemical safety evaluation that includes adding metabolic competence to *in vitro* HTS (Thomas et al., 2019; EPA, 2021).

An important component of toxicity evaluations is modeling functional liver effects to simulate first-pass hepatic metabolism and distal target tissue exposure to circulating metabolites. *In vitro* tools commonly used for recapitulating hepatic metabolic reaction pathways include use of subcellular fractions, genetically engineered cells, hepatoma cell lines, stem cell-derived hepatocytes, and primary hepatocytes (Ooka et al., 2020; Serras et al., 2021). Hepatic S9 subcellular fractions have historically been used as a metabolizing system in the Ames mutagenicity test (Maron and Ames, 1983; Hakura et al., 2003; OECD, 2020) and contain phase I and II metabolic enzymes present in microsomal and cytosolic fractions (Parmentier et al., 2006). However, direct application of exogenous S9 can cause issues including cytotoxicity via formation of toxic microsomal lipid peroxides (Tan et al., 1982; Cox et al., 2016), potentially altered toxicological activity (Heringa et al., 2004; Kwon et al., 2020), or technical interference (Dreier et al., 2002). Strategies to avoid the technical limitations of S9 fractions in HTS include direct mRNA transfection to transiently express cytochrome P450 enzymes (DeGroot et al., 2018), use of microsomal fractions (Ooka et al., 2022), or incorporation of recombinant metabolizing enzymes (Yu et al., 2018). S9-associated assay interference can be mitigated by encapsulation in alginate-based hydrogel microbeads (Yamamoto et al., 2011).

Inspired by the pillar plate design used previously to encapsulate cells in alginate microspheres (Yu et al., 2018), the alginate immobilization of metabolic enzymes (AIME) method (Deisenroth et al., 2020; Hopperstad et al., 2022) encapsulates S9 fractions in alginate microspheres immobilized on plastic pillar lid inserts to support phase I hepatic metabolism in assay medium supplemented with a nicotinamide adenine dinucleotide phosphate-regenerating system (NADPH) (Stanley, 2017). Applications of the lid-based AIME method have successfully evaluated metabolism-dependent false positive and false negative target assay effects in HTS studies (Deisenroth et al., 2020; Hopperstad et al., 2022), with some technical limitations. Cofactors for phase II metabolism were not included, so parent chemicals that yield functional metabolites strictly via phase II metabolism were not identified. Further, the use of custom fabricated lids limits assay throughput and method transfer capabilities for broader adoption.

The objectives of this study were to address workflow limitations and improve transferability of the AIME method by adapting the concept to an automated bioprinting platform and incorporating liquid-handling instrumentation for increased speed and precision. Further, the addition of requisite cofactors for phase II metabolism expanded the metabolic capacity of the AIME method compared to earlier versions that only supported phase I metabolism. Metabolic competence of the bioprinting method was first assessed using enzymatic activity assays. Then, like previous studies involving the lid-based AIME method (Deisenroth et al., 2020; Hopperstad et al., 2022), the bioprinting method was combined with the OECD Test Guideline 455 VM7Luc estrogen receptor transactivation (ERTA) assay to estimate transformation-related effects. A training set of 25 chemicals that previously exhibited metabolism-dependent shifts in estrogenic bioactivity was selected. Additional refinement of the AIME bioprinting method has the potential to expand HTS capabilities in a robust, accessible manner to integrate *in vitro* xenobiotic metabolic competence into routine screening workflows.

## 2 Methods

### 2.1 Development of bioprinter AIME method

The AIME method involves the encapsulation of S9 fractions in alginate microspheres and application to parent compounds for metabolic transformation supported by a cofactor for phase I metabolism, nicotinamide adenine dinucleotide phosphate (NADPH). This method has been applied in a lid-based format consisting of custom manufactured 384-well microplate pillar lids (Supplementary Figure S1) that are functionalized with Matrigel, dipped into an S9-alginate mixture, and then dipped into crosslinking solutions to form microspheres attached to solid supports. Detailed methods for the lid-based method are described in Deisenroth et al. (2020). Here, the AIME method was adapted to a bioprinter (Cellink BIOX, Gothenburg, Sweden) and additional cofactors were incorporated to support phase II metabolism, including uridine 5′-diphosphoglucuronic acid trisodium salt (0.5 mM UDPGA) for glucuronidation, glutathione (0.5 mM GSH) for glutathione conjugation, and adenosine 3′-phosphate 5′-phosphosulfate lithium salt hydrate (2 µM PAPS) for sulfation (Richardson et al., 2016; Stanley, 2017). A reagents list for all procedures is available in Supplementary Table S1.

A series of preliminary experiments determined optimal bioprinting parameters using a syringe pump printhead programmed to deposit S9-alginate directly into 384-well microplates (Supplementary Table S2). An automated liquid handler (Agilent MultiFlo FX Dispenser, Santa Clara, CA) crosslinked alginate using 40 µL/well 33 mM barium chloride and 0.4% poly-L-lysine solution, and then rinsed microspheres using 80 µL/well 1X phosphate buffered saline, resulting in a prepared plate containing alginate microspheres in designated wells.

#### 2.1.1 Comparison of lid and bioprinter AIME methods

The lid- and bioprinter-based AIME methods were directly compared using a P450-Glo CYP3A4 luminescent assay (Promega, Madison, WI) and phase I cofactor NADPH with and without S9-alginate microspheres. Opti-MEM I Reduced-Serum Medium (ThermoFisher, Waltham, MA) was charged using an NADPH regeneration system (NRS). Following NADPH generation, charged medium was diluted 1:11.4 with uncharged medium. Luciferin IPA was added to a final concentration of 3 μM S9-alginate microspheres were produced using the lid- and bioprinter-based methods, and NADPH-supplemented medium was added to wells (40 μL/well). Assays proceeded for 2 hours at 37°C and 5% CO_2_ followed by the addition of Luciferin Detection Reagent (LDR) with esterase (40 μL/well). Assay plates were equilibrated to room temperature (20°C–22°C) for 20 min and read on a CLARIOstar microplate reader (BMG Labtech Inc., Cary, NC) using a luminescent endpoint protocol. Raw data were normalized to control wells and expressed as relative light unit fold-change (FC). Data were analyzed using GraphPad Prism version 9.4.1, and statistical significance (*p* ≤ 0.05) was determined using a Welch’s two sample *t*-test (*n* = 4).

#### 2.1.2 Estimation of phase I and II metabolic activity

The bioprinting method was next evaluated using additional CYP enzymes to confirm broad activity in the presence of cofactors for both phase I and II metabolism. Medium was charged with NADPH as described in Section 2.1.1 and supplemented with phase II cofactors UDPGA, GSH, and PAPS. Promega P450-Glo substrates luciferin-IPA (3 µM), -ME (100 µM), -H (100 µM), and -2B6 (3 µM) were used to measure CYP3A, CYP1A, CYP2C, and CYP2B activity, respectively. Cofactor-supplemented media was added to wells (40 μL/well) with and without bioprinted S9-alginate microspheres. Assays proceeded for 2 hours at 37°C and 5% CO_2_ followed by the addition of corresponding LDR (40 μL/well) as described in assay kit protocols. Assay plates were read and data analyzed using methods described in Section 2.1.1 (*n* = 4).

Uridine diphospho-glucuronosyltransferase (UGT) activity was measured to estimate phase II biotransformation in the presence of cofactors, including UDPGA, using a commercial UGT Activity Assay (BioVision, Milpitas, CA). UGT substrate was reconstituted in DMSO and diluted to a 1X final concentration in either phase I and II cofactor-supplemented UGT assay buffer (+UDPGA) or unsupplemented UGT assay buffer (-UDPGA) in plates containing bioprinted S9-alginate microspheres (100 µL/well total volume). The UGT positive control and standard curve were prepared according to the assay protocol without the addition of microspheres. Fluorescence was measured with a CLARIOstar microplate reader (Ex/Em = 415/502 nm) in kinetic mode with reads every 5 min for 120 min at 37°C. Data were normalized to the 0 min timepoint, and statistical significance was determined using a two-way ANOVA with a Greenhouse-Geisser correction using GraphPad Prism (*n* = 3).

### 2.2 Application of bioprinter AIME method to estrogen receptor transactivation assay

#### 2.2.1 Estrogen receptor transactivation assay with metabolic competence

The VM7Luc4E2 stable cell line (formerly BG1Luc4E2) (Rogers and Denison, 2000; Li et al., 2014) is a variant of the MCF7 human breast cancer cell line that contains an estrogen receptor (ER) responsive luciferase reporter gene to evaluate the transactivation potential of endogenous hERa, and to a lesser extent hERb, in response to estrogenic test substances (OECD, 2016). The VM7Luc4E2 cell line was maintained with routine passaging and estrogen-stripped for assay seeding as described previously (Deisenroth et al., 2020). VM7Luc4E2 cells were seeded into white 384-well microplates (Greiner Bio-One, Monroe, NC) at 2.5 × 10^4^ cells in a total volume of 30 µL/well using a Certus Flex Micro Dispenser (Fritz Gyger AG, Bern, Switzerland) and acclimated for 18–24 h in an incubator set at 37°C and 5% CO_2_ prior to exposure to test chemicals.

Chemicals exhibiting large metabolism-dependent shifts were selected from previous AIME studies (Deisenroth et al., 2020; Hopperstad et al., 2022). Test chemicals solubilized in DMSO to a final concentration of 100 mM were either obtained in Echo qualified 384 PP polypropylene source microplates (Labcyte, San Jose, CA) from the ToxCast library (Branford, CT), or were solubilized in-house, and were stored at −80°C in a desiccator prior to use (Supplementary Table S3). Plate-based controls run on each assay plate were consistent with Hopperstad et al. (2022) and include: 17β-estradiol (100p.m.) for ER-activation, trans-Stilbene (100 µM) for metabolism-dependent ER-activation, ethylparaben (100 µM) for metabolism-dependent ER-inactivation, and DMSO (0.2%) as a solvent control. Parent source plates containing the top solubilized concentration were used to generate daughter source plates in Echo-qualified 384 PP microplates using an Echo 555 acoustic liquid handler (Labcyte, San Jose, CA). Daughter source plates were sealed and stored at room temperature (20°C–22°C) and protected from light for the duration of the screen.

A 9-point dilution series with 2X concentrations (0.004–400 µM) skewed toward the upper half of the series was generated by dispensing test chemicals and backfilling with a complementary volume of DMSO using Echo Cherry Pick software (v.1.6.2) to a final dispensed volume of 400 nL. Cofactor-supplemented ERTA assay medium (100 µL/well) (Supplementary Table S1) was dispensed into 21 test columns (0.2% [v/v] DMSO final concentration) using a Certus Flex Micro Dispenser. Untreated assay medium was dispensed (100 µL/well) into remaining columns. Plates were mixed on a Multi-Microplate Genie mixer (Scientific Industries, Bohemia, NY) at 500 rpm for 10 min. Metabolism occurred at 2X concentrations, and the resulting solution was added 1:1 to cells, resulting in a final 1X test compound concentration range that included: 2.49 nM, 160.5 nM, 1.245 µM, 4.98 µM, 14.95 µM, 33.6 µM, 67 µM, 119.5 µM, and 199 µM.

A ViaFlo 384-channel semiautomated pipette (Integra Bioscience, Hudson, NH) was used to transfer 70 µL of dosed medium to freshly prepared AIME plates containing metabolically inactive (dH_2_O-alginate, Met Neg) microspheres in rows 2-8 and metabolically active (S9-alginate, Met Pos) microspheres in rows 9–15. Assay plates were incubated for 2 hours at 37°C and 5% CO_2_ to facilitate metabolic transformation, and then 30 µL of conditioned medium containing parent compounds and metabolites was transferred to assay plates containing estrogen-stripped VM7Luc4E2 cells. Following incubation, 30 µL/well of reconstituted Bright-Glo Luciferase Assay reagent (Promega, Madison, WI) was added. Plates were incubated for 5 minutes at room temperature, and then a luminescent endpoint was read on a CLARIOstar microplate reader. Each test chemical concentration series was screened as a single technical replicate for each experimental replicate (*n* = 4).

#### 2.2.2 Data modeling and analysis

Raw concentration-response data from the ERTA assay were fit and analyzed using the ToxCast Data Analysis Pipeline (TCPL v.3.0.0; Judson et al., 2022) (Filer et al., 2017), which utilizes an updated curve-fitting and hit-calling package (tcplfit2 v.0.1.3) (Sheffield et al., 2021) in R v.4.2.1 (R Core Team, 2022). Well-level data were obtained as raw luminescence units. Baseline values (*bval*) were calculated as the plate-level median of the NRS-supplemented DMSO control. Response values with low well quality as determined by Echo 555 acoustic liquid handler reports were removed to generate corrected response values (*cval*). *Cval* was normalized to zero-centered fold-change using the following equation:

$$resp=\frac{cval}{bval}-1$$

where *resp* is the normalized response variable for model fitting. Each chemical concentration-response series was fit to ten models (constant, linear, quadratic, power, hill, gain-loss, exponential 2, exponential 3, exponential 4, exponential 5), with the winning model determined through the minimum of the Akaike Information Criteria (AIC). The efficacy cut-off was set to three times the baseline median absolute deviation (bmad), which was defined as the median absolute deviation of the normalized cofactor-supplemented DMSO control *resp* values. Tcplfit2 utilizes a probabilistic “continuous hit call” to quantify the strength of hits and identify borderline cases (Sheffield et al., 2021). Classification criteria for hit calls were defined as: 0 = negative, 0 > and <0.9 = equivocal, ≥0.9 = positive. Summary performance metrics for the control compounds were determined for assay variability (coefficient of variation, CV) and screening quality (Z′-factor) (Zhang et al., 1999).

Designation of estrogenic effects were determined by continuous hit calls from the TCPL curve fits. The area under the curve (AUC) for winning models was determined using the trapezoidal rule (pracma v.2.3.8; Borchers, 2022). The difference in AUC between winning curve fits for assay modes with and without metabolism defined the directional shift trends where bioinactivation <0, no change = 0, and bioactivation >0. Efficacy (maximum median response) and potency (benchmark response dose, BMD) were calculated with TCPL for both metabolism modes.

## 3 Results

### 3.1 Development of bioprinter AIME method

**FIGURE 1:**
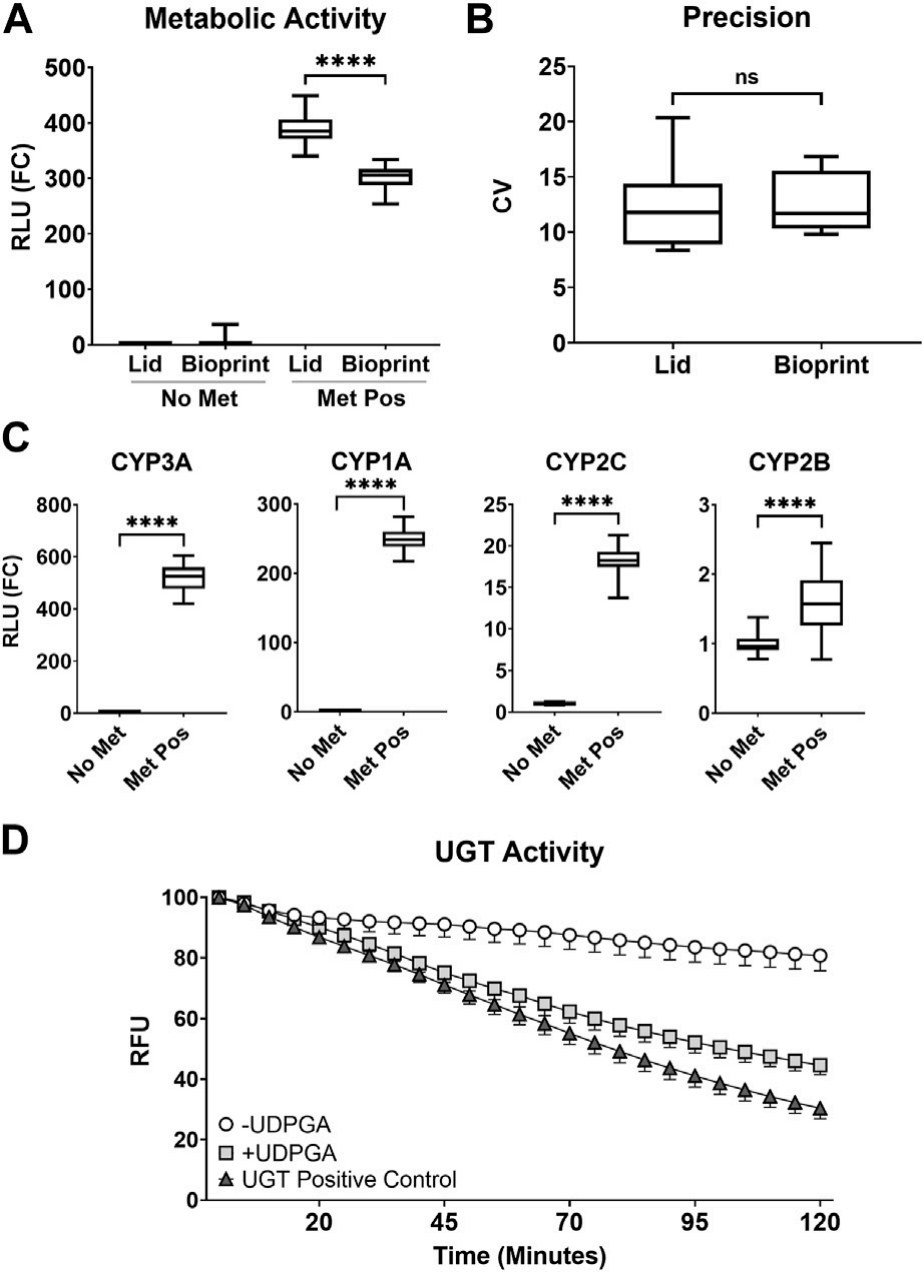
**(A)** Comparison of CYP3A metabolic activity for the AIME lid-based (Lid) and bioprinting (Bioprint) methods. Assay without metabolism (No Met, no microspheres), assay with metabolism (Met Pos, S9-alginate microspheres), and relative light unit fold-change (RLU (FC)) are shown. The summary distributions represented in the box and whisker plots for **(A–C)** are the 2.5th percentile, 25th percentile, median, 75th percentile, and 97.5th percentile (**** = *p* < 0.0001). **(B)** Inter-well precision between the AIME lid-based (Lid) and bioprinting (Bioprint) methods based on coefficient of variation (CV) (No significance = ns). **(C)** Activity of common phase I CYP enzymes (**** = *p* < 0.0001). **(D)** Temporal evaluation of phase II glucuronidation kinetics (UGT Activity) in the absence (-UDPGA, white circle) and presence (+UDPGA, light grey square) of uridine 5′-diphosphoglucuronic acid trisodium salt (UDPGA), shown in comparison to the UGT Positive Control (dark grey triangle). Data are expressed as % control relative light units (%RLU). Error bars represent 95% confidence intervals around the mean.

The AIME bioprinting method was adapted from the previously published lid-based method, and the two methods were compared in a P450-Glo CYP3A4 luminescent assay to determine dynamic range and variation. The lid-based method demonstrated higher mean activity (388.6 ± 27.1 FC) compared to the bioprinting method (300.5 ± 22.6 FC) (*p* < 0.0001, Figure 1A). However, there was no significant difference in inter-well variation between the lid-based (12.32 ± 3.95 FC) and bioprinting (12.66 ± 2.71 FC) methods (*p* = 0.845, Figure 1B).

A broad panel of metabolic activity was characterized for the bioprinting method, including activity of CYP enzymes CYP3A, CYP1A, CYP2C, and CYP2B. All CYP enzymes showed a significant increase in activity in the presence of S9-alginate (Met Pos) compared to no-metabolism (No Met) controls (*p* < 0.0001, Figure 1C), supporting the conclusion that phase I metabolism occurred in the bioprinting platform.

Lack of phase II metabolism was a noted limitation in prior versions of the lid-based method (Deisenroth et al., 2020; Hopperstad et al., 2022). Here, assay medium was supplemented with cofactors to support phase I and II metabolism, and the AIME bioprinting method was assessed for a prominent phase II metabolic pathway, glucuronidation. A commercial UGT Activity Assay utilized a highly fluorescent UGT substrate which decreases in fluorescence emission as the substrate was converted into a non-fluorescent glucuronide conjugate. A significant difference between treatments with and without metabolic cofactors was observed (*p* < 0.0001); the cofactor-supplemented treatment exhibited a greater drop in fluorescence emission compared to the negative control (Figure 1D). This indicates the UGT substrate was converted into a non-fluorescent glucuronide conjugate more rapidly in the presence of metabolic cofactors, and that the AIME bioprinting method is competent for glucuronidation. Additional cofactors for sulfation and glutathione conjugation were included to further support phase II metabolic pathways but were not evaluated.

### 3.2 Application of bioprinter AIME method to estrogen receptor transactivation assay

**FIGURE 2:**
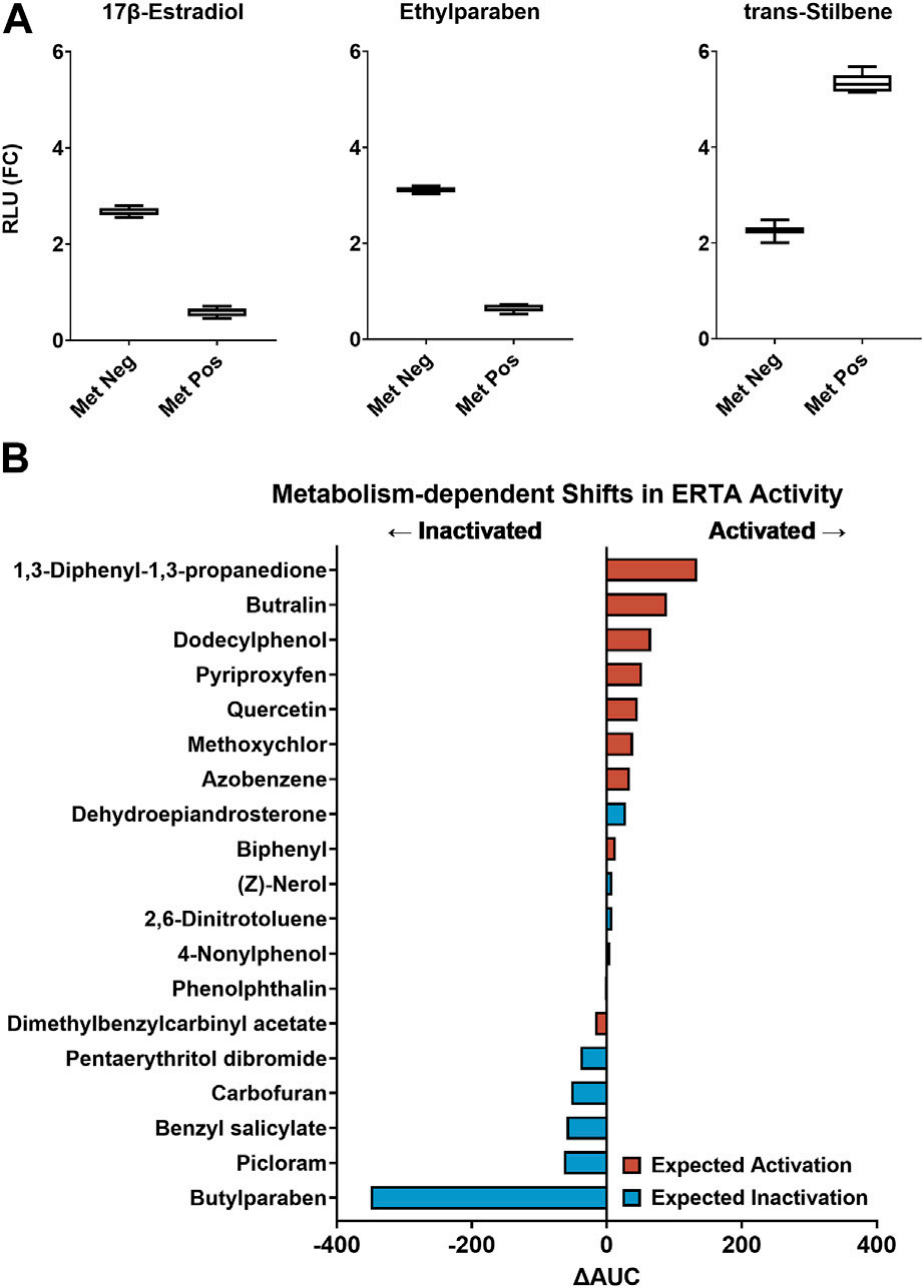
**(A)** Performance of the VM7Luc ERTA assay plate-based controls: ERTA positive control (17β-estradiol), metabolism bioinactivation control (ethylparaben), and metabolism bioactivation control (trans-Stilbene) in metabolism-negative (Met Neg, dH_2_O-alginate microspheres) and metabolism-positive (Met Pos, S9-alginate microspheres) modes are expressed as relative light units fold-change (RLU (FC)). The summary distributions represented in the box and whisker plots are the 2.5th percentile, 25th percentile, median, 75th percentile, and 97.5th percentile. **(B)** 19 estrogenic chemicals rank-ordered by ∆AUC for evaluation of metabolism-dependent bioactivity. Zero-center is represented by a solid line. A negative ∆AUC value indicates a decrease in estrogenic activity with metabolism (←Inactivated) and a positive ∆AUC value indicates an estrogenic activity increase with metabolism (Activated→). Expected transformations are based on previous AIME-ERTA studies and are symbolized for activation (red) and inactivation (blue).

The VM7Luc ERTA assay was implemented in a proof-of-concept experiment to determine the ability of the AIME bioprinting method to accurately classify chemicals previously characterized as having strong metabolism-dependent ER effects (Deisenroth et al., 2020; Hopperstad et al., 2022). Reference chemicals included 17β-estradiol as the positive reference standard for the ERTA endpoint, ethylparaben as the metabolism bioinactivation control, trans-Stilbene as the metabolism bioactivation control, and DMSO as the solvent control. Compounds were tested with and without cofactor supplementation in the presence of dH_2_O-alginate (“metabolism-negative”) and S9-alginate (“metabolism-positive”) microspheres. Reference chemicals exhibited expected metabolism-dependent directional shifts in activity: with metabolism, mean efficacy was reduced for 17β-estradiol (−78.0%) and ethylparaben (−79.2%), and trans-Stilbene demonstrated increased activity (+136.1%). Directional shifts were consistent with previous versions of the assay and indicative of metabolic competence with bidirectional biotransformation of estrogenic reference chemicals (Figure 2A) (Deisenroth et al., 2020; Hopperstad et al., 2022).

Assay variability and screening quality performance metrics were calculated for reference chemicals in metabolism-positive mode (containing S9-alginate microspheres), in metabolism-negative mode (containing dH_2_O-alginate microspheres), and for DMSO-baseline conditions (containing no microspheres) (Table 1). CV is indicative of variability, and values did not deviate considerably from DMSO-baseline conditions (≤16.75%) except for 17β-estradiol and ethylparaben in metabolism-positive mode. As expected, the Z′-factor values for all conditions were in an acceptable range (0.49–0.61), except for 17β-estradiol and ethylparaben in metabolism-positive mode (0.20 and 0.14) where metabolic inactivation decreased the separation between positive and negative modes. Importantly, Z′-factor values were greater than 0.5 for conditions designed to evaluate parent chemical (17β-estradiol; metabolism-negative) and metabolite(s) (ethylparaben and trans-stilbene; metabolism-positive) bioactivity, indicating metabolism proceeded as expected.

**TABLE 1:** Assay variability (coefficient of variation; CV) and screening quality (Z′-factor), for negative control (DMSO), ERTA positive control (17β-estradiol), metabolism bioinactivation control (ethylparaben), and metabolism bioactivation control (trans-Stilbene).

Compound	Metabolism	CV	Z'-factor
DMSO	No Met	10.98	NA
17β-estradiol	Met Neg	12.78	0.61
	Met Pos	14.93	0.20
Ethylparaben	Met Neg	10.70	0.51
	Met Pos	16.75	0.14
trans-Stilbene	Met Neg	10.68	0.49
	Met Pos	10.96	0.52

Performance parameters were evaluated for AIME assay metabolism modes including baseline conditions (No Met, no microspheres), metabolism-negative (Met Neg, dH_2_O-alginate microspheres), and metabolism-positive (Met Pos, S9-alginate microspheres). Not applicable (NA).

TCPL data modeling identified chemicals with estrogenic activity in the ERTA assay, and differences in AUC (ΔAUC) of model curve fits estimated shifts in bioactivity between metabolism modes (Supplementary Table S4). Of the 25 chemicals tested, 22 were classified as ER-active in at least one mode of the ERTA assay (88%), and 17 were concordant with results from previous AIME studies (68%). Based on TCPL hit calls, complete bioinactivation of benzyl salicylate and butylparaben was observed with the addition of metabolic competence, while bioactivation was observed for biphenyl and pyriproxyfen (Table 2). Eighteen chemicals were active in both assay modes. When ranked by ΔAUC for metabolism-dependent bioactivity, both activation and inactivation trends were noted (Figure 2B). The magnitude of ΔAUC values indicates the difference in potency and efficacy between metabolism-positive and negative assay modes. Chemicals with ΔAUC values near zero exhibited marginal shifts between assay modes (e.g., (Z)-nerol; 2,6-dinitrotoluene) indicating estrogenic activity was not markedly different with metabolism (Figure 2B; Supplementary Figure S2). Considering chemicals screened for biotransformation that displayed estrogenic activity (19 total), 5/8 (62.5%) were concordant with expected bioinactivation, and 9/11 (81.8%) with expected bioactivation. Overall, 14/19 (73.7%) were concordant with expected biotransformations (Table 2; Figure 2B).

**TABLE 2:** Estrogenic activity (hit call, hitc), efficacy (represented by maximum median response value, max_med [FC]), and potency (represented by benchmark response dose values, BMD [µM]) are reported for the AIME-ERTA assay in metabolism-negative (Met Neg, dH_2_O-alginate microspheres) and metabolism-positive (Met Pos, S9-alginate microspheres) assay modes.

Chemical name	Classification	Concordance	Estrogenic activity (hitc)		Efficacy (max_med)		Potency (bmd)	
			Met Neg	Met Pos	Met Neg	Met Pos	Met Neg	Met Pos
Daidzein	Agonist	1	1.00	1.00	4.57	5.78	0.22	0.41
Resveratrol	Agonist	1	1.00	1.00	3.22	4.32	4.54	9.12
Atrazine	Agonist Negative	0	1.00	1.00	0.77	0.56	101.02	113.58
Spironolactone	Agonist Negative	1	0.00	0.66	0.43	0.23	>200	NA
1,3-Diphenyl-1,3-propanedione	Bioactivated	1	1.00	1.00	1.64	3.45	4.39	4.86
2-Nitrobenzenamine	Bioactivated	0	0.00	0.00	0.10	0.08	>200	>200
4-Nonylphenol	Bioactivated	1	1.00	0.99	0.68	0.81	2.88	2.63
Azobenzene	Bioactivated	1	1.00	1.00	1.05	3.41	27.10	29.79
Biphenyl	Bioactivated	1	0.07	1.00	0.56	0.62	>200	116.90
Butralin	Bioactivated	1	1.00	1.00	0.83	2.13	9.11	7.56
Dimethylbenzylcarbinyl acetate	Bioactivated	0	1.00	1.00	1.04	1.11	58.23	107.96
Dodecylphenol	Bioactivated	1	1.00	1.00	1.43	1.78	0.90	0.45
Methoxychlor	Bioactivated	1	1.00	1.00	0.99	1.66	1.28	1.02
Phenolphthalin	Bioactivated	0	1.00	1.00	1.52	1.90	44.02	56.39
Pyriproxyfen	Bioactivated	1	0.42	1.00	1.13	1.73	NA	6.76
Quercetin	Bioactivated	1	1.00	1.00	1.72	2.32	22.22	11.61
(Z)-Nerol	Bioinactivated	0	1.00	1.00	0.48	0.54	135.87	71.00
2,6-Dinitrotoluene	Bioinactivated	0	1.00	1.00	0.77	1.08	79.95	58.34
Benzyl salicylate	Bioinactivated	1	1.00	0.04	1.70	0.53	12.06	NA
Butylparaben	Bioinactivated	1	1.00	0.88	4.92	0.38	3.14	180.06
Carbofuran	Bioinactivated	1	1.00	1.00	1.27	0.62	19.12	118.90
Dehydroepiandrosterone	Bioinactivated	0	1.00	1.00	2.48	2.52	0.20	0.23
Penoxsulam	Bioinactivated	0	0.00	0.00	0.04	0.04	>200	>200
Pentaerythritol dibromide	Bioinactivated	1	1.00	1.00	1.81	1.36	12.08	24.29
Picloram	Bioinactivated	1	1.00	1.00	1.96	2.22	4.56	29.88

Classification represents expected metabolic transformations based on previous AIME-ERTA studies. Concordance represents the comparison between observed ∆AUC-based shifts and classifications, where 0 = not concordant and 1 = concordant.

## 4 Discussion

The incorporation of xenobiotic metabolic competence into HTS has the potential to decrease uncertainty associated with *in vitro* extrapolation to potential human health effects. An existing NAM, the lid-based AIME method, has successfully evaluated metabolism-dependent target assay effects in HTS studies (Deisenroth et al., 2020; Hopperstad et al., 2022), with some technical limitations. These limitations were addressed by adapting the AIME concept to a bioprinting platform, expanding phase II enzyme metabolic competence, and automating the workflow. The resulting AIME bioprinting method produced significant CYP activity with similar precision to the previously published lid-based method but exhibited lower assay sensitivity (Figures 1A–C). Phase II metabolic competence was successfully incorporated to the AIME method by the addition of relevant cofactors, and glucuronidation, a well-recognized phase II metabolic pathway, was observed (Figure 1D).

After demonstrable metabolic activity was confirmed by biochemical assays, the AIME bioprinting method was deployed in a proof-of-concept ERTA assay to evaluate metabolism-dependent ER effects for a set of previously evaluated compounds. Assay performance was determined for reference compounds with and without metabolism to ensure that the assay was robust and reproducible across experimental runs. By design, Z′-factor values shifted accordingly for the bioinactivated and bioactivated reference controls in a metabolism-dependent manner, indicating metabolic transformation was measurable with the bioprinting method. Partial inactivation of the positive steroid control, 17β-estradiol, was also evident, as previously observed (Hopperstad et al., 2022). CV values indicated experimental errors were within acceptable ranges (below 20%). These observations reemphasize the importance of incorporating reference compounds for the target bioassay and metabolic platforms.

In this study, 15/19 test compounds were classified as estrogenic with or without metabolism, meaning most of the ER-active chemicals would have been identified without metabolic transformation. However, directional shifts due to biotransformation may be informative—a weakly estrogenic parent compound bioactivated to strongly estrogenic metabolite(s), or the contrary, is useful information when prioritizing hazard effects. Metabolic shifts in the AIME bioprinting method were calculated based on the difference in AUC for fitted curves between metabolism-negative and metabolism-positive assay modes (Figure 2A; Supplementary Figure S2). The ΔAUC metric classified twelve chemicals as bioactivated and seven as bioinactivated, demonstrating concordance with 81.8% and 62.5% of expected shifts, respectively. Compound classifications that were inconsistent with expected metabolic classifications were marginally shifted and may be less of a priority for toxicological relevance. Such relevance could be further considered with statistical testing but this wasn’t employed in this study as shifts in AUC were only used to rank metabolism-dependent effects. Alternatively, inconsistencies may be due to phase II biotransformation reactions altering parent chemicals or metabolic intermediates to better approximate *in vivo* biotransformation-related effects compared to previous evaluations that used phase I cofactors only.

It is important to note the overall magnitude of normalized ER activity in both metabolism modes was markedly lower than previous lid-based AIME studies. This, coupled with the observation of lower activity in a direct comparison between the lid-based and bioprinting methods indicates that some feature(s) of the bioprinting method are contributing to reduced sensitivity. A limitation of this study was that the relative amount of S9 encapsulated for each method wasn’t quantified; sensitivity may have been impacted by the degree of enzymatic activity as it related to S9 quantity and kinetics. It is also possible that lower observed rates of metabolism may have resulted from a change in alginate microsphere size and/or morphology (Uyen et al., 2020). Additional evaluation of microsphere size, bioprinter dispensing technique, and incubation time may increase sensitivity reflective of the lid-based method, and application of the bioprinter method to different assay types can inform the breadth of method compatibility. Such refinements of the AIME bioprinting method have the potential to expand HTS capabilities in a robust, accessible manner to incorporate *in vitro* xenobiotic metabolic competence.

## Data Availability

The original contributions presented in the study are included in the article/Supplementary Material, further inquiries can be directed to the corresponding author.
