# Supplementary material for: Development of a bioprinter-based method for incorporating metabolic competence into high-throughput in vitro assays
Source: Front Toxicol. 2023 May 4;5:1196245. doi: 10.3389/ftox.2023.1196245 (PMC10192685; doi:10.3389/ftox.2023.1196245)
Supplement: Supplementary file 1 [file Image1.PDF]

Supplementary Material

Development of a Bioprinter-based Method for Incorporating Metabolic Competence into High-throughput *In Vitro* Assays

Kristen Hopperstad, Chad Deisenroth*

*** Correspondence:** Chad Deisenroth: deisenroth.chad@epa.gov

Supplementary Figure 1. Image of custom manufactured 384-well microplate pillar lid type utilized in lid-based AIME method. Pillars were functionalized with Matrigel, dipped into an S9-alginate mixture, and then dipped into 0.1 M barium chloride and 0.01% poly-L-lysine crosslinking solutions to form microspheres attached to solid supports.

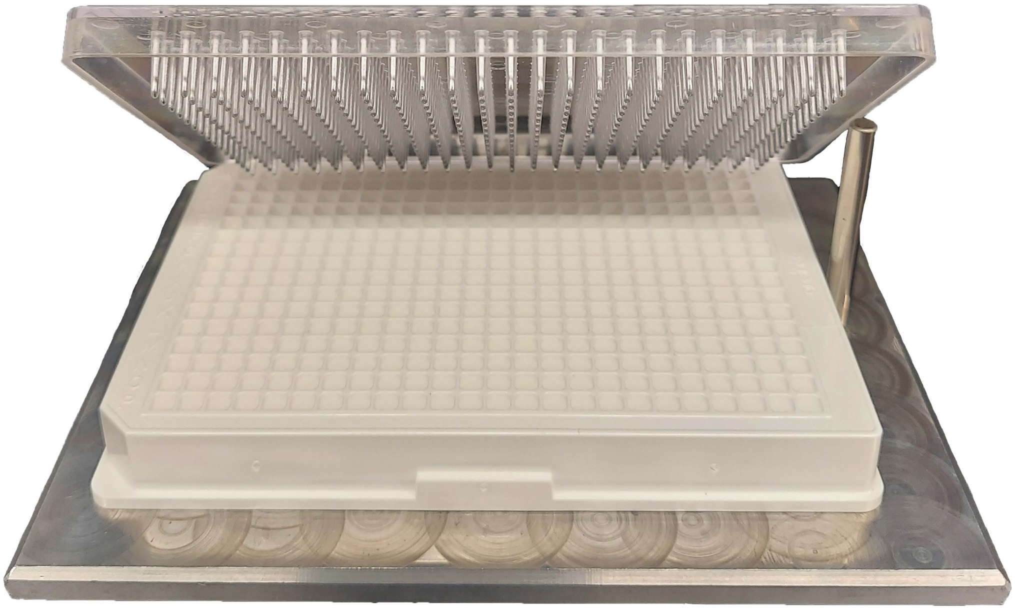

Supplementary Material

Supplementary Figure 2. Winning TCPL model curve fits for metabolism-negative (solid blue, dH_2O-alginate microspheres) and metabolism-positive (solid red, S9-alginate microspheres) ERTA assay modes. Plots are shown for all compounds screened in the ERTA assay. Horizontal lines indicate the efficacy cutoff (3*bmad) for metabolism-negative (dashed blue) and metabolism-positive (dashed red) modes. Compounds were screened in a 9-point titration series (0.002-200mM) and are represented as log10 concentration (logc) μM. Data points are zero-centered normalized responses (resp) plotted for all experimental replicates (n = 4).

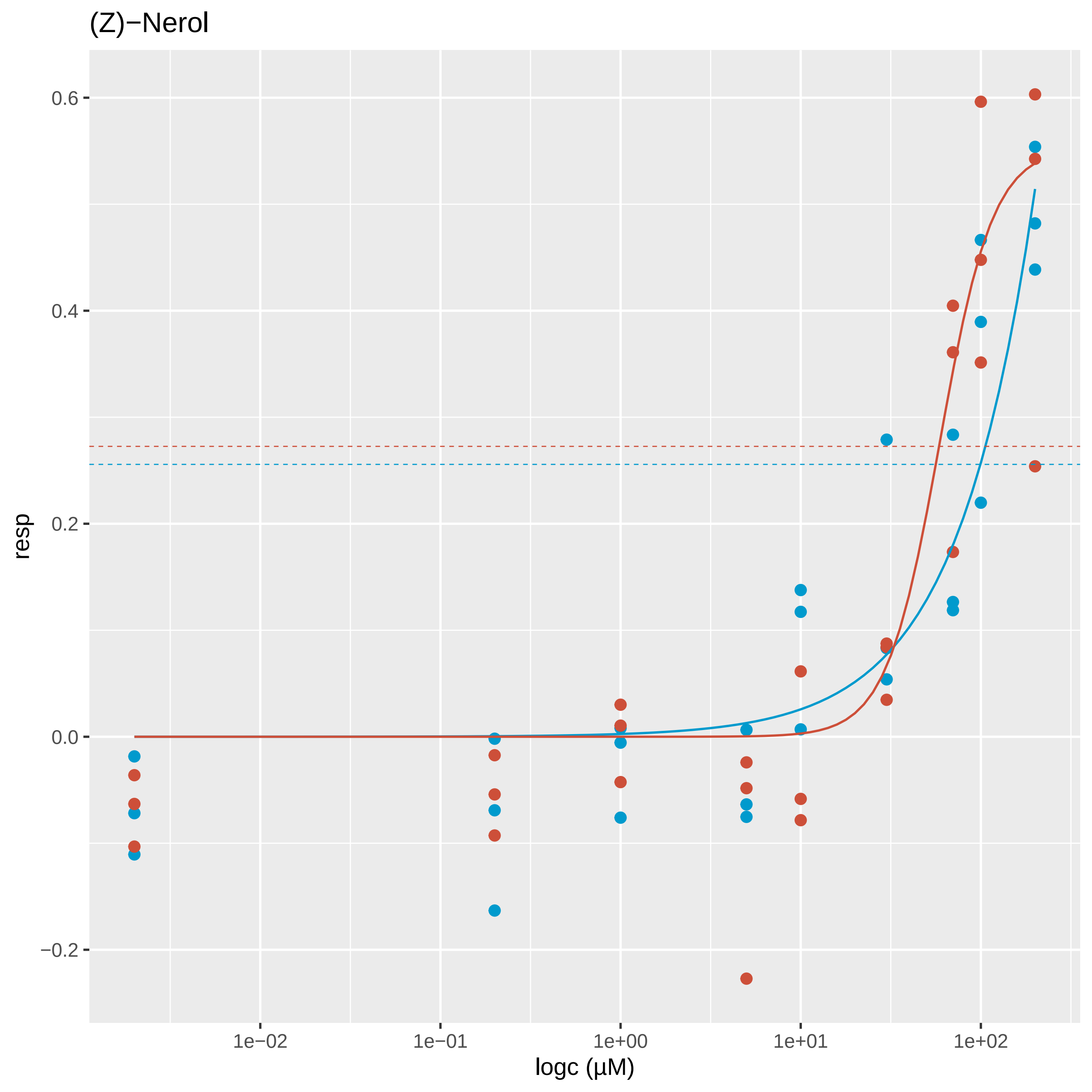

(Z)−Nerol
0.6
0.4
0.2
0.0
−0.2
resp
1e−02
1e−01
1e+00
1e+01
1e+02
logc (μM)

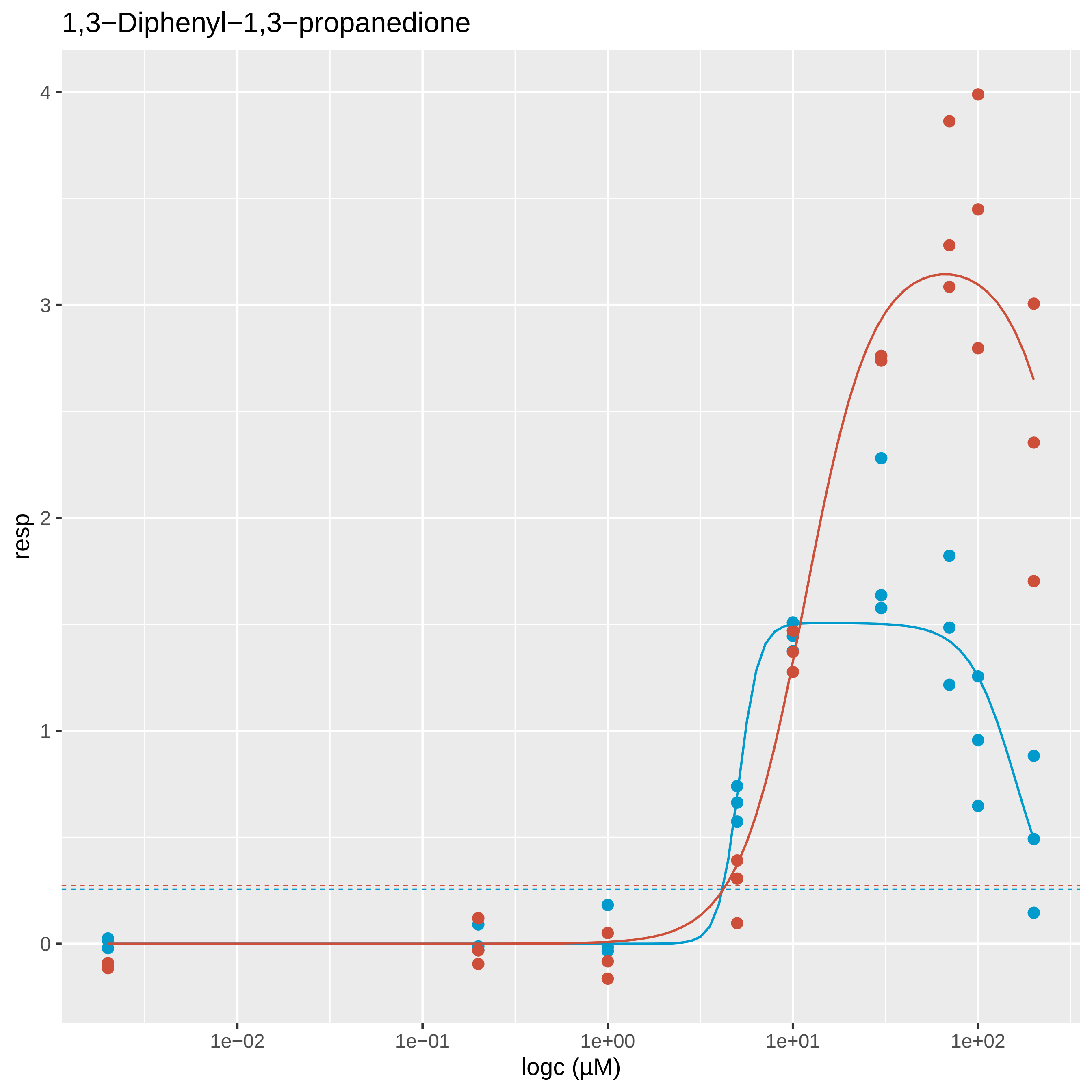
1,3−Diphenyl−1,3−propanedione
resp
logc (μM)
0
1
2
3
4
1e−02
1e−01
1e+00
1e+01
1e+02

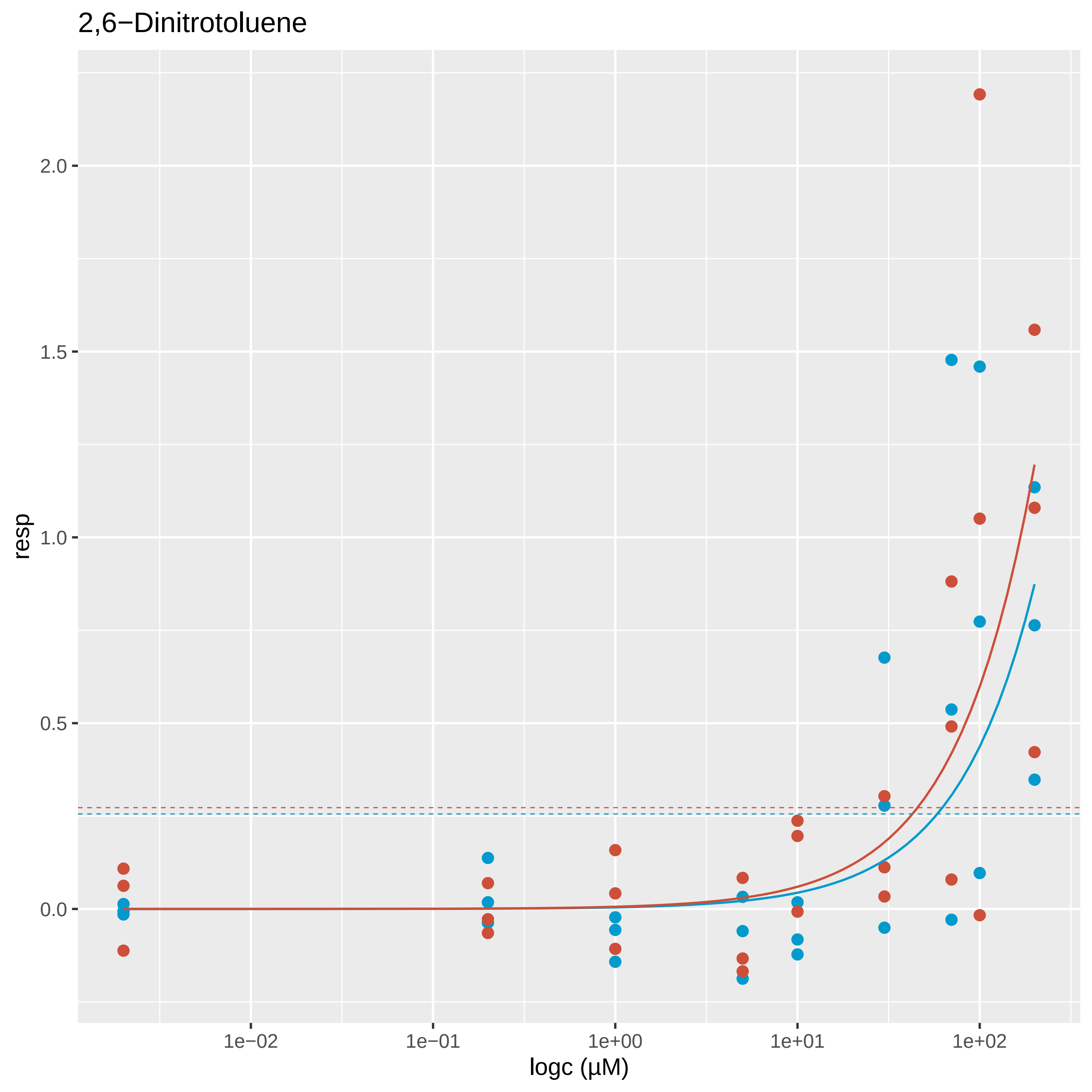

2,6−Dinitrotoluene
resp
logc (µM)
2.0
1.5
1.0
0.5
0.0
1e−02
1e−01
1e+00
1e+01
1e+02

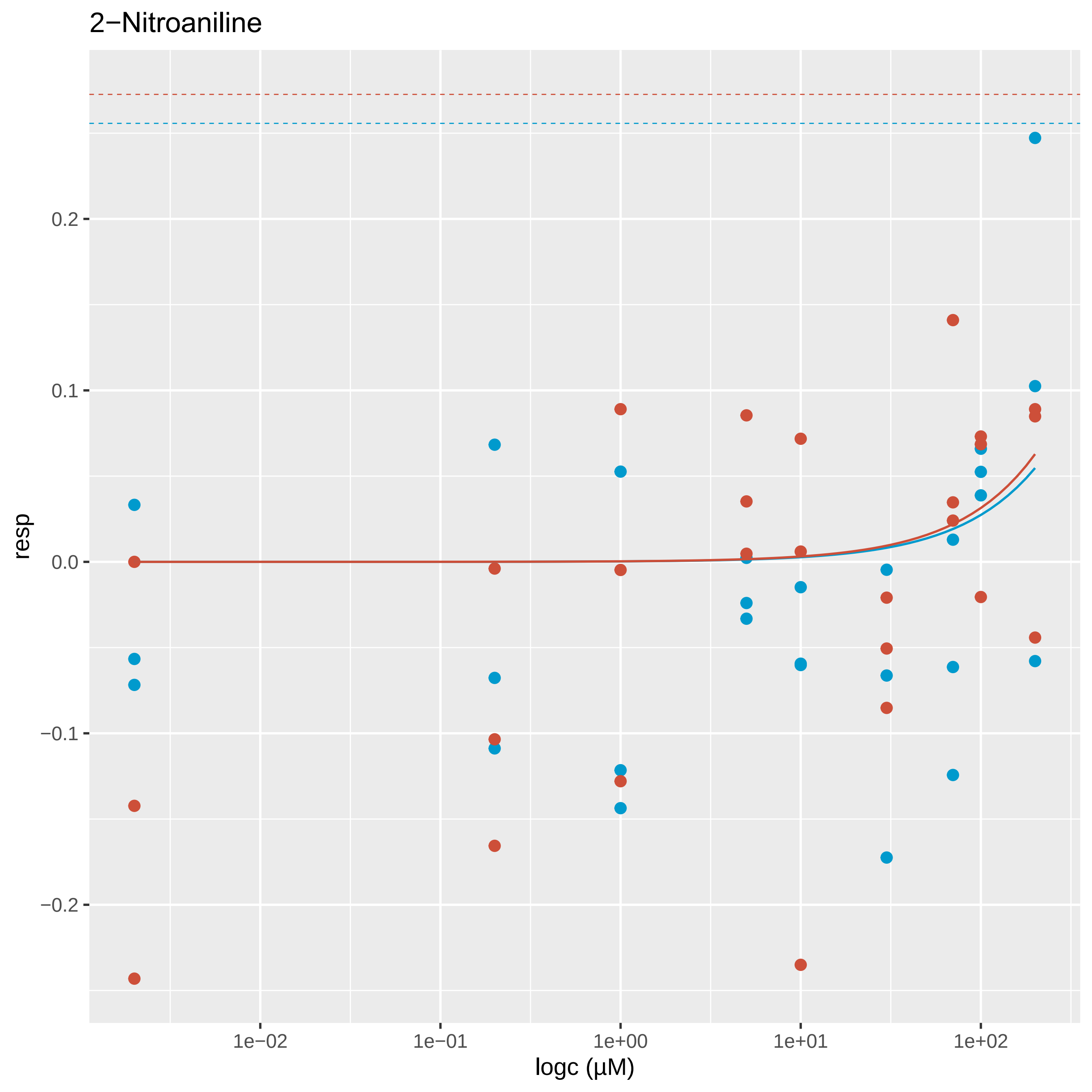

2−Nitroaniline
resp
logc (µM)
0.2
0.1
0.0
−0.1
−0.2
1e−02
1e−01
1e+00
1e+01
1e+02

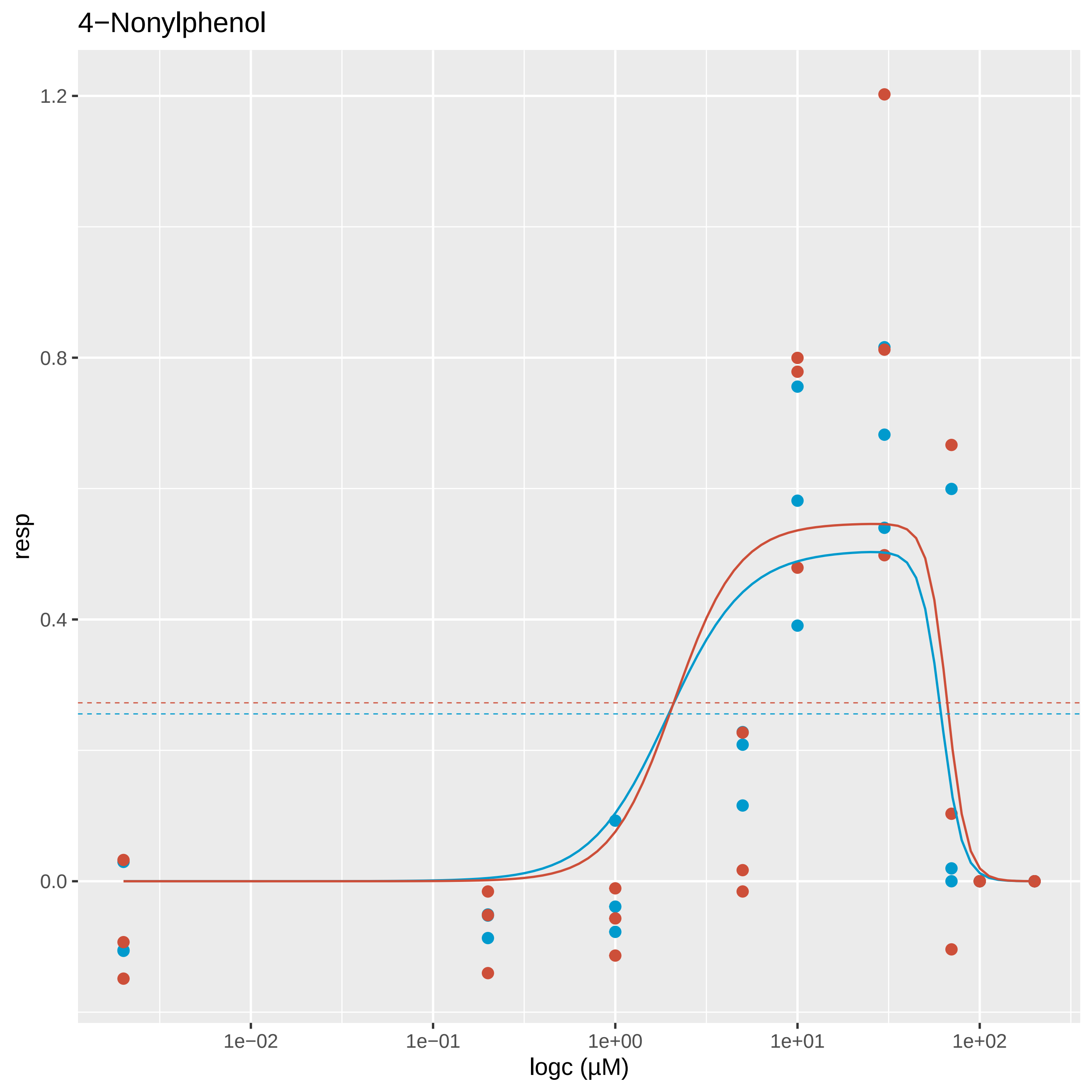
4−Nonylphenol
resp
1.2
0.8
0.4
0.0
1e−02
1e−01
1e+00
1e+01
1e+02
logc (µM)

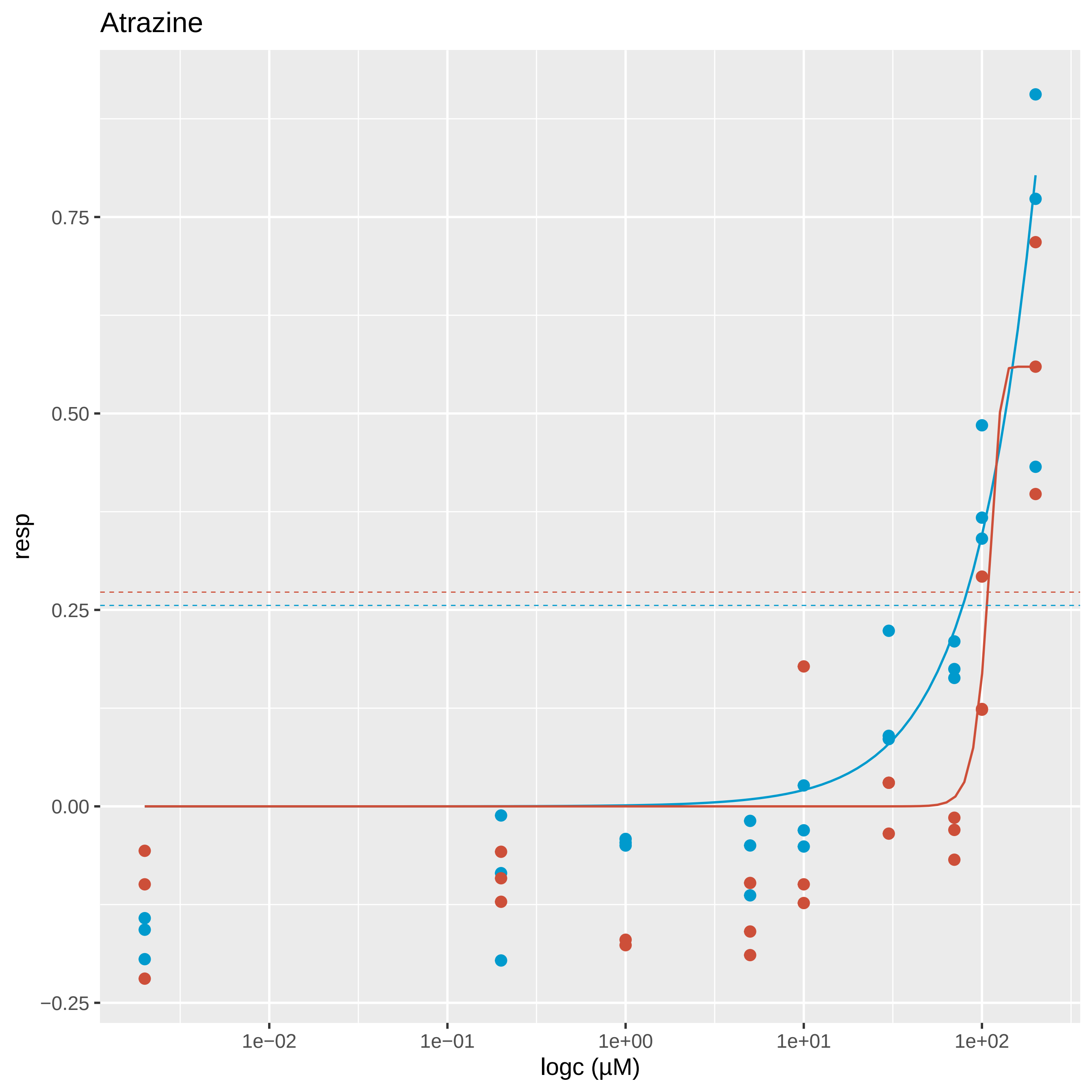
Atrazine
resp
0.75
0.50
0.25
0.00
−0.25
1e−02
1e−01
1e+00
1e+01
1e+02
logc (µM)

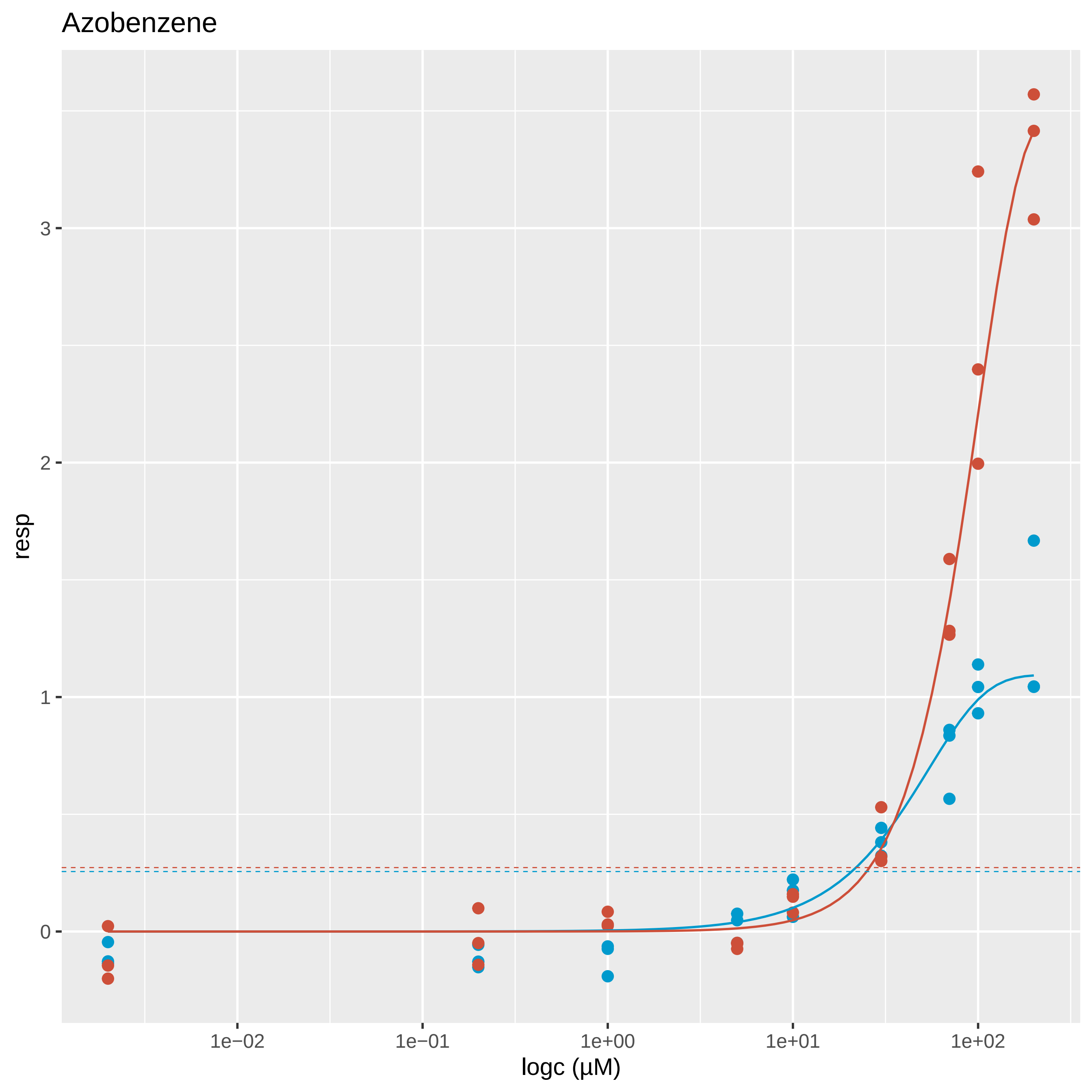

Azobenzene
resp
3
2
1
0
1e−02
1e−01
1e+00
1e+01
1e+02
logc (µM)

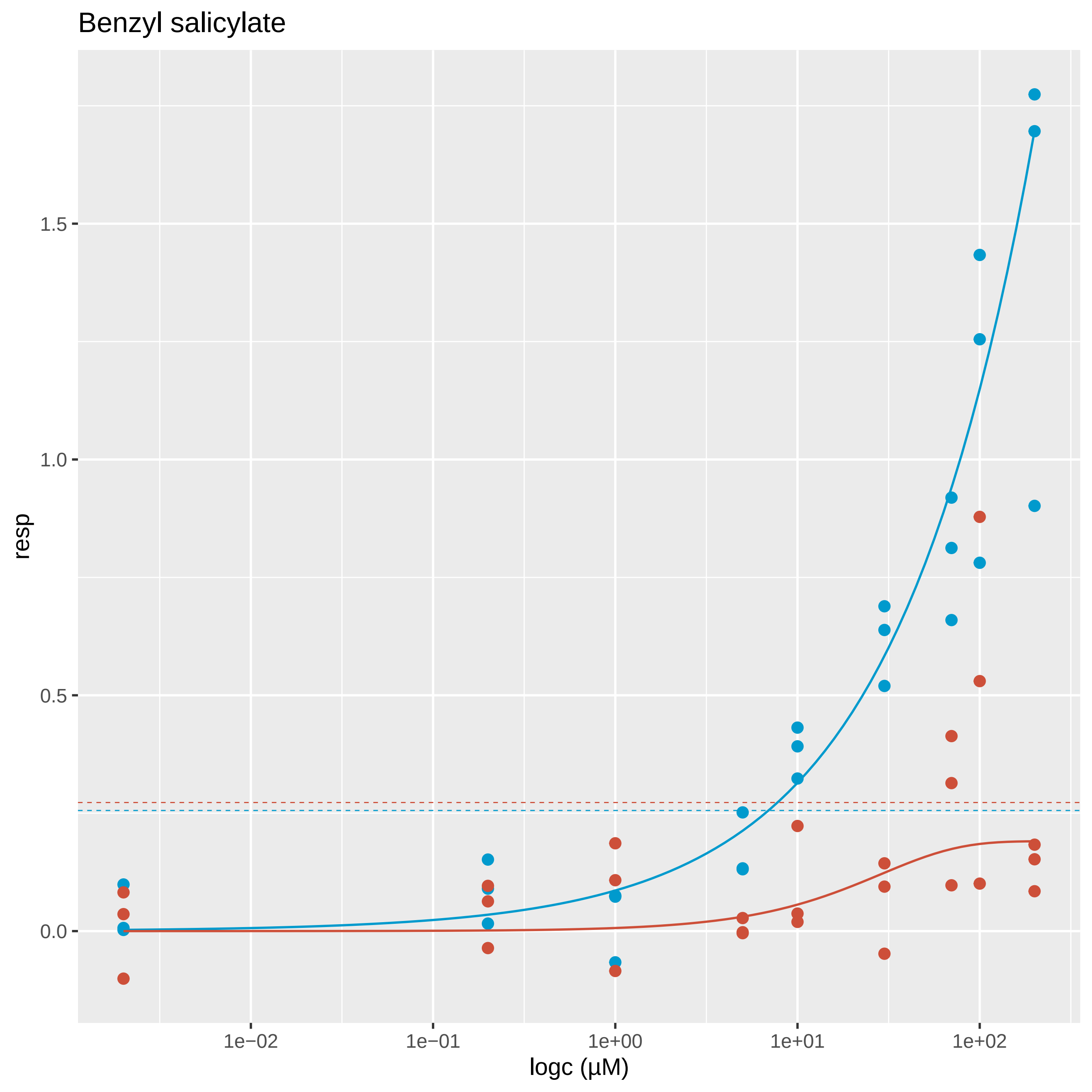
Benzyl salicylate
resp
logc (µM)
1.5
1.0
0.5
0.0
1e−02
1e−01
1e+00
1e+01
1e+02

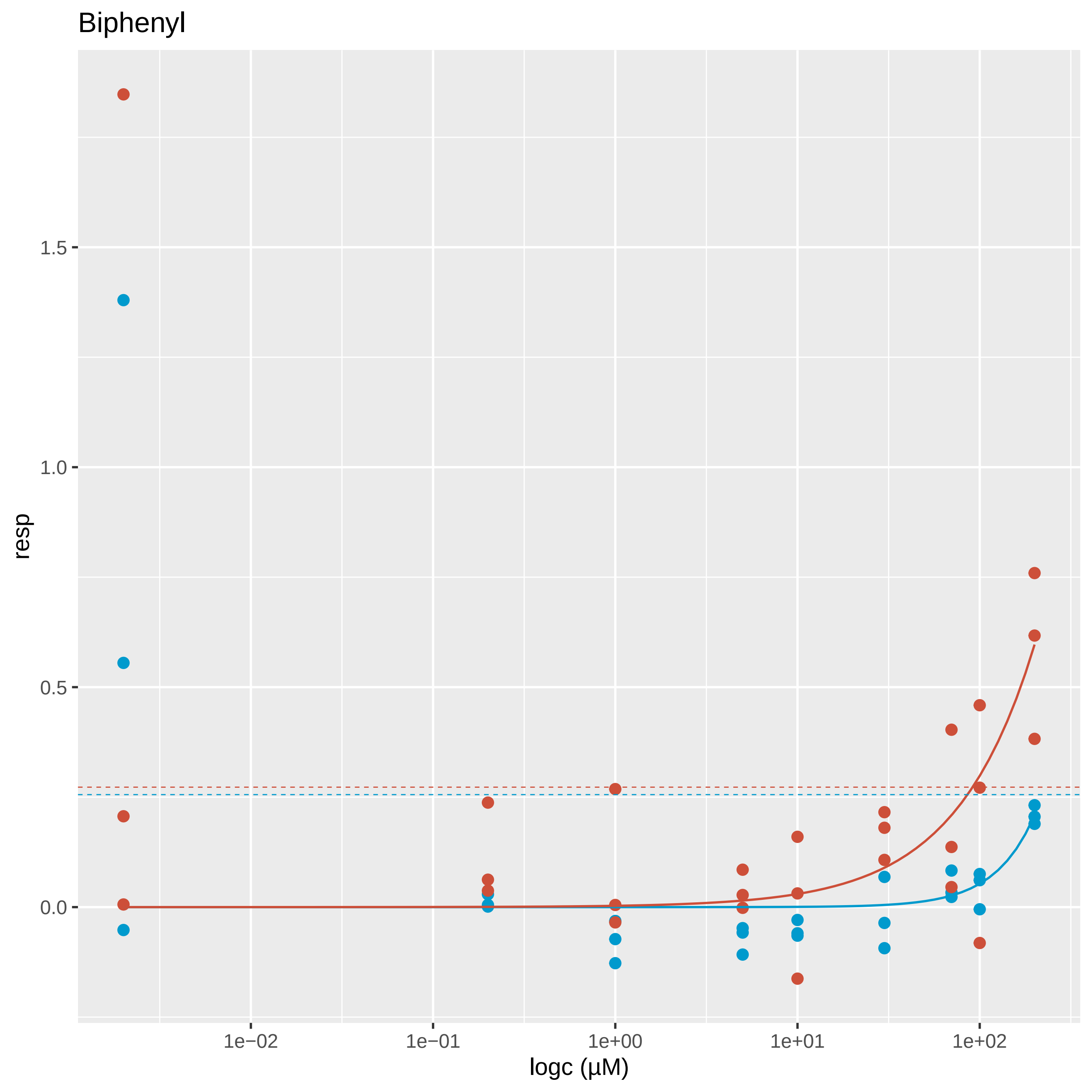

Biphenyl
resp
1.5
1.0
0.5
0.0
1e−02
1e−01
1e+00
1e+01
1e+02
logc (µM)

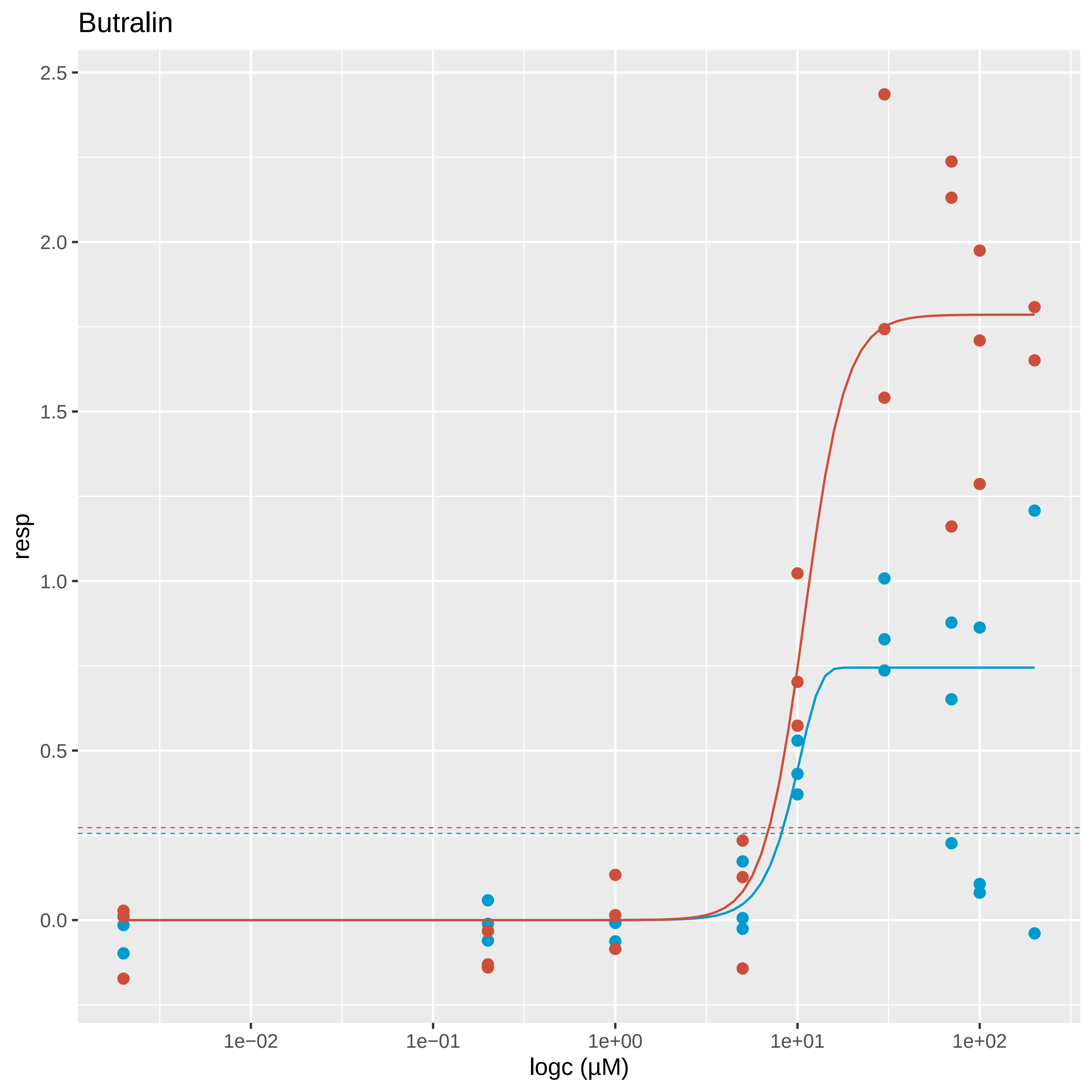

Butralin
2.5
2.0
1.5
1.0
0.5
0.0
resp
1e−02
1e−01
1e+00
1e+01
1e+02
logc (μM)

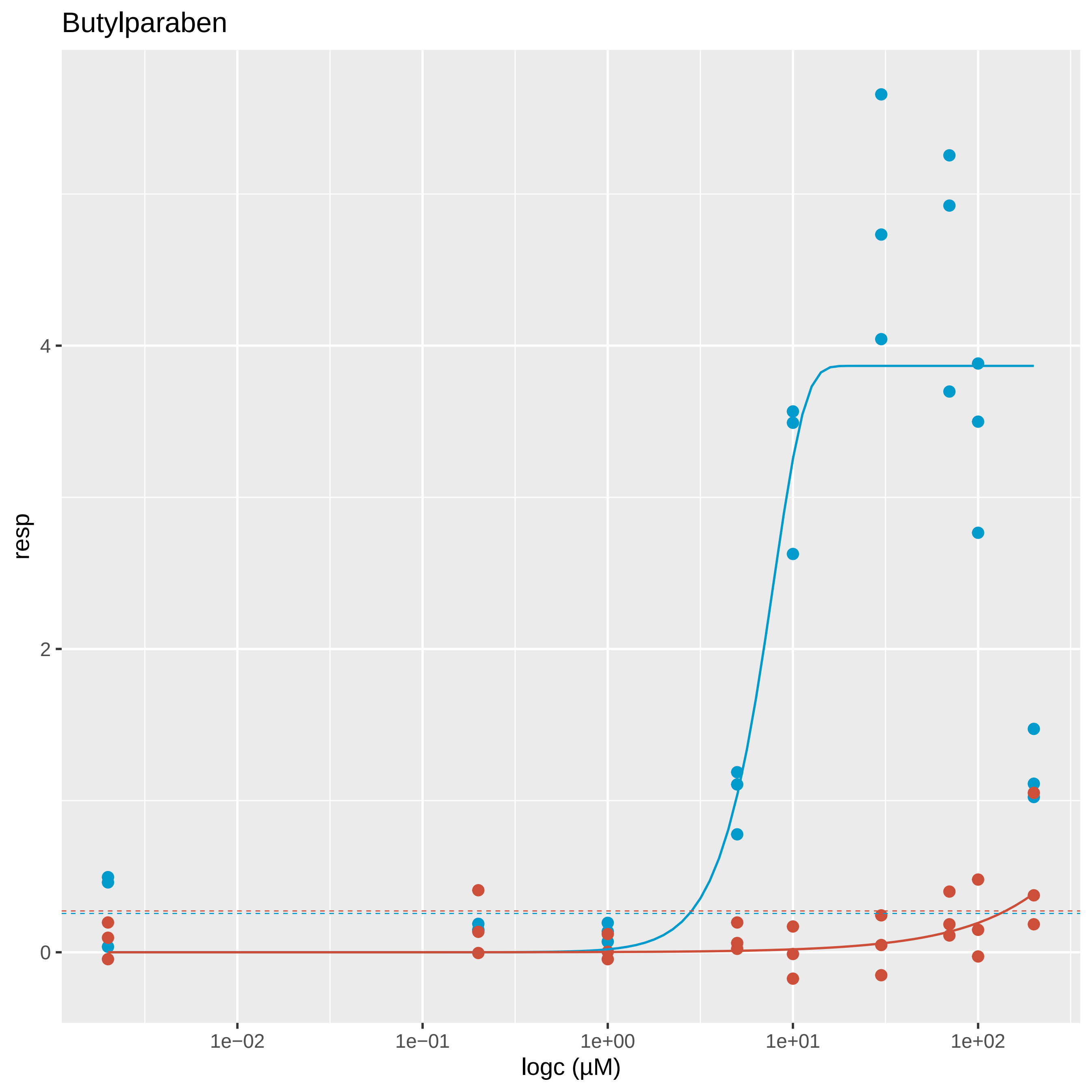
Butylparaben
resp
4
2
0
1e−02
1e−01
1e+00
1e+01
1e+02
logc (µM)

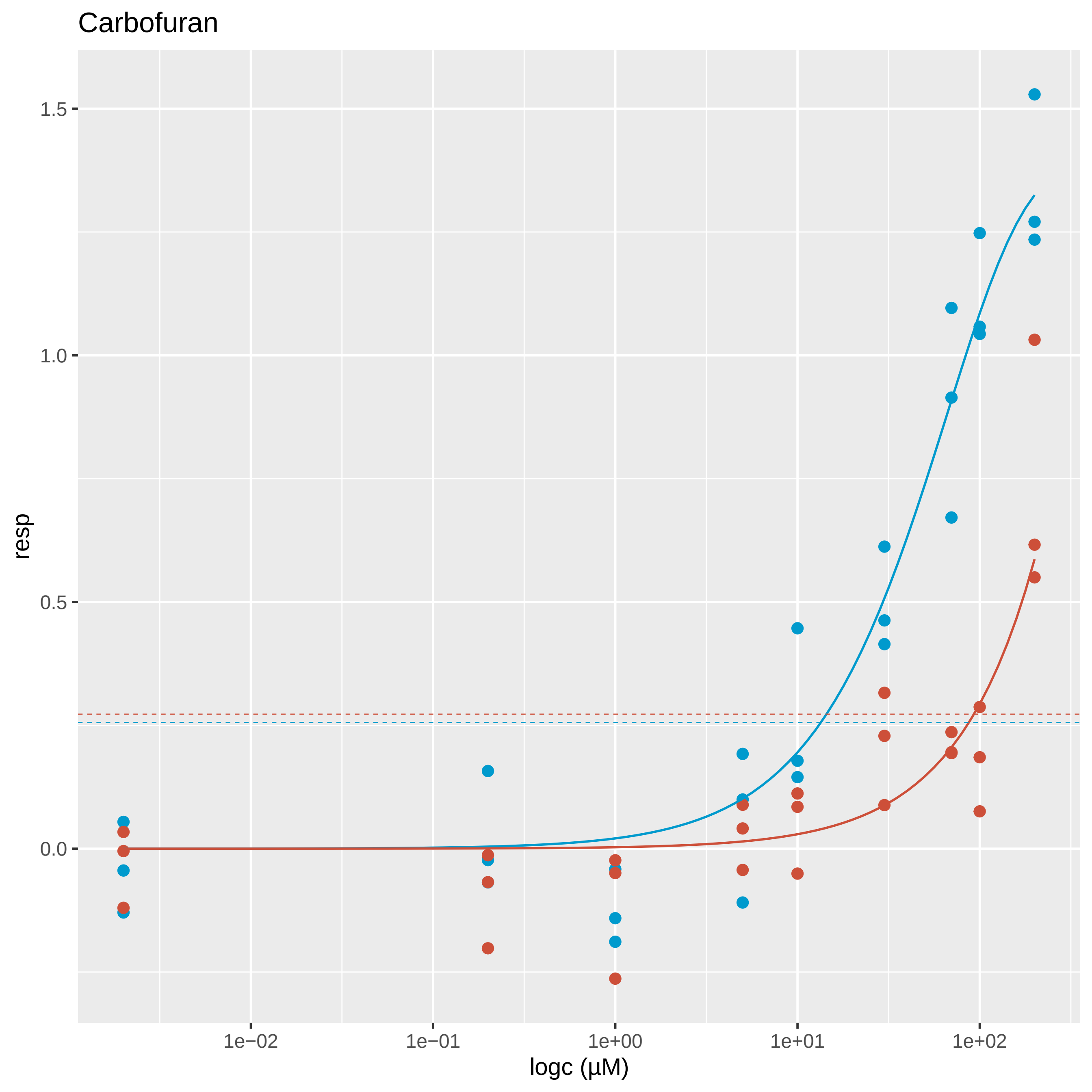
Carbofuran
resp
1.5
1.0
0.5
0.0
1e−02
1e−01
1e+00
1e+01
1e+02
logc (µM)

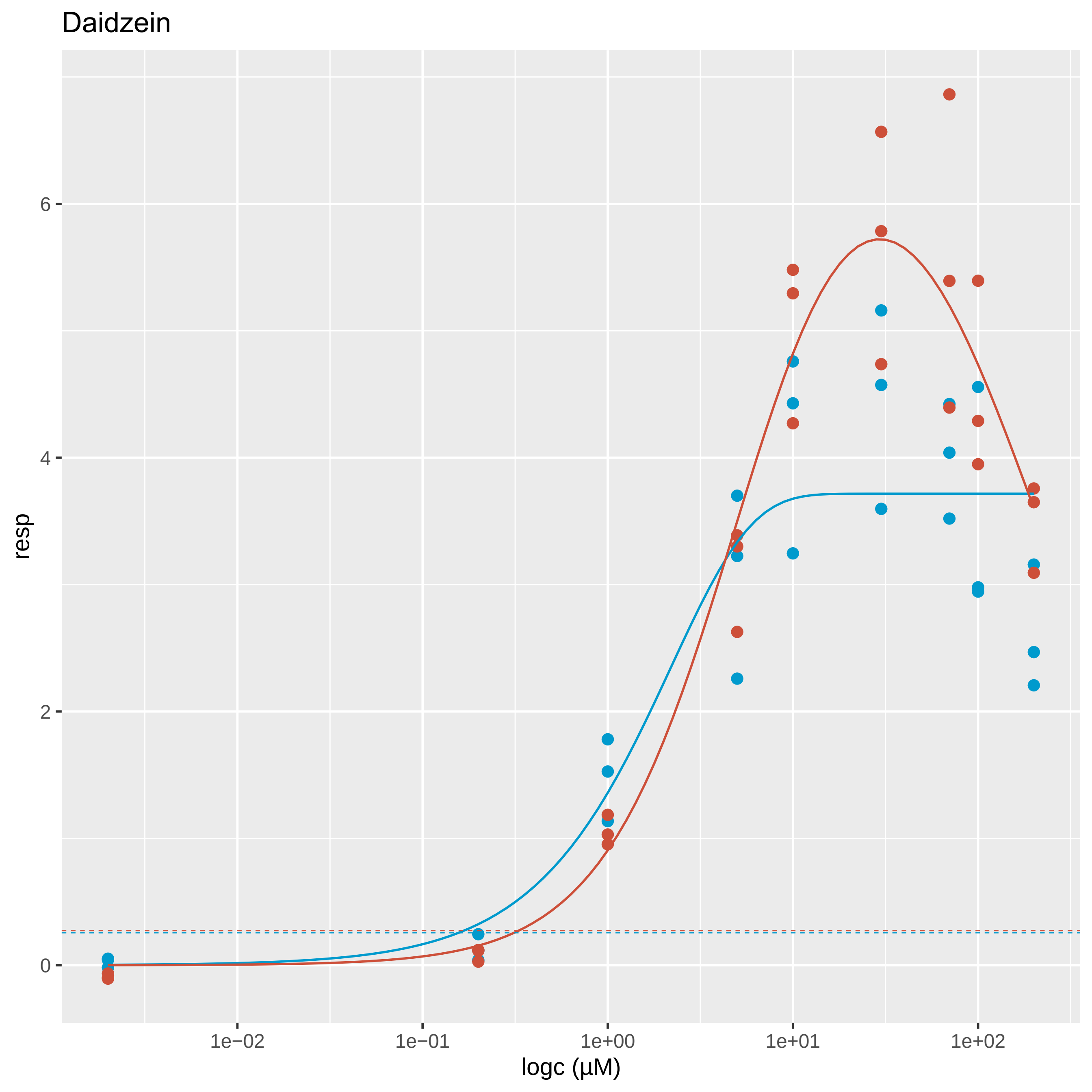
Daidzein
resp
6
4
2
0
1e−02
1e−01
1e+00
1e+01
1e+02
logc (µM)

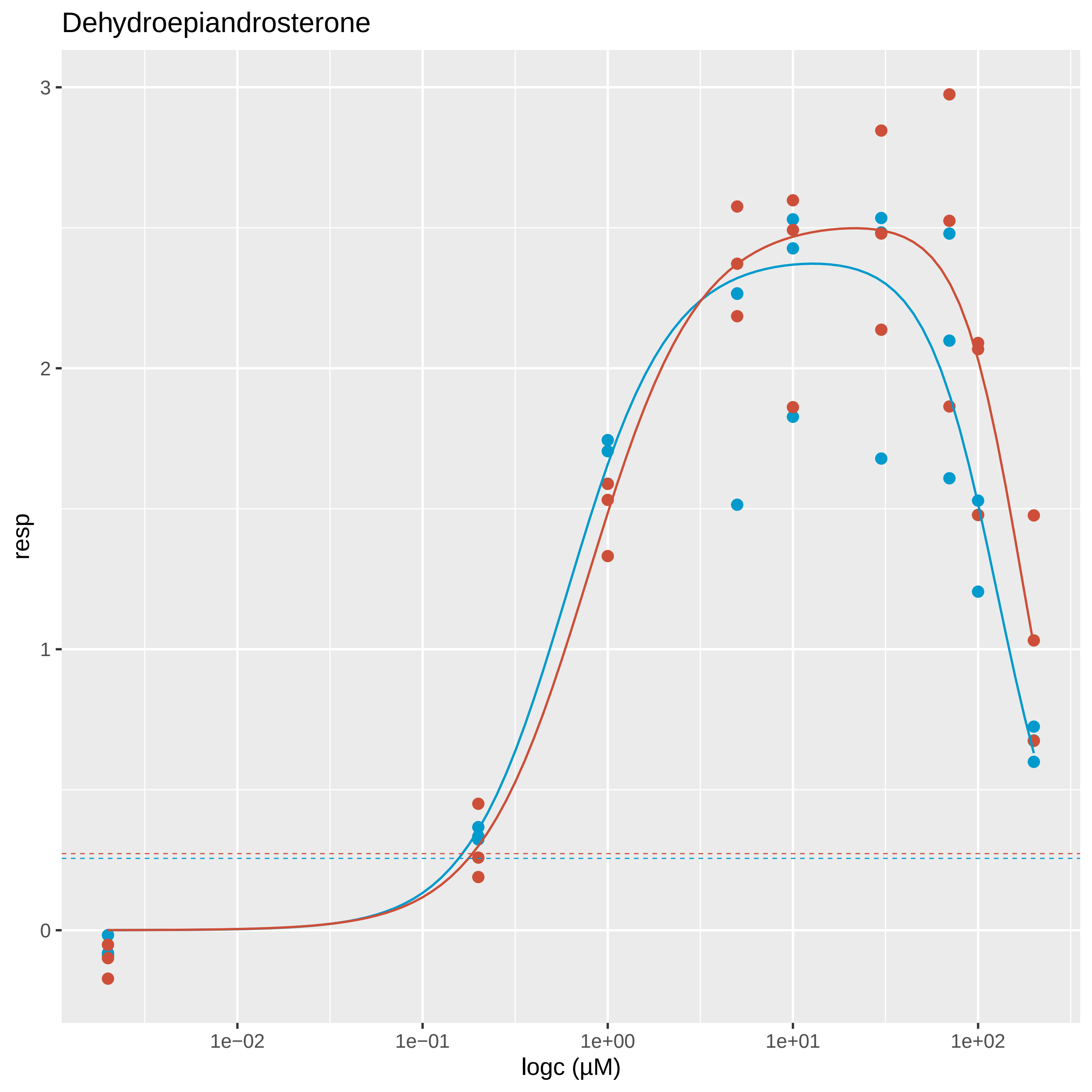

Dehydroepiandrosterone
resp
3
2
1
0
1e−02
1e−01
1e+00
1e+01
1e+02
logc (µM)

Dimethylbenzylcarbinyl acetate

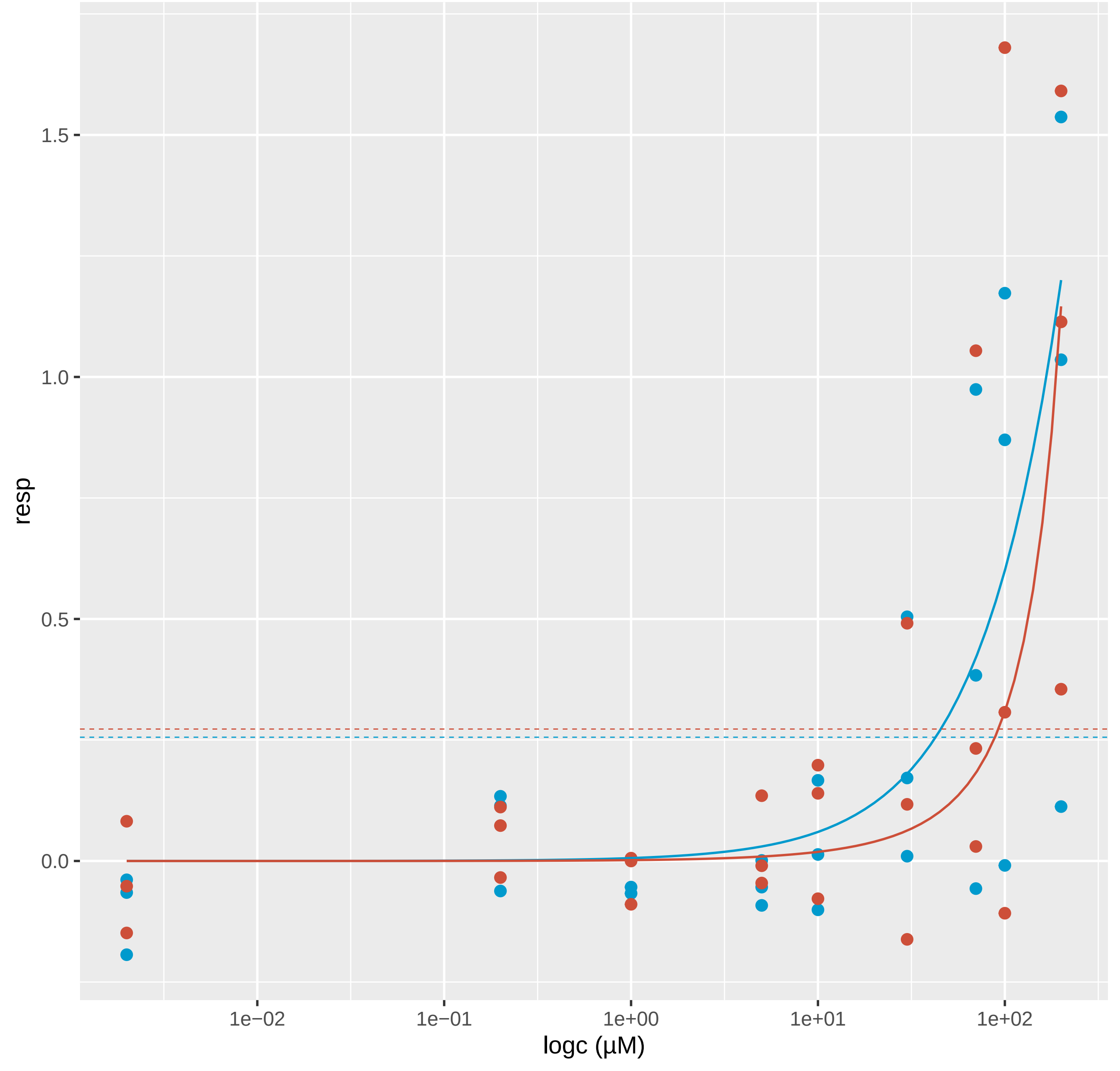

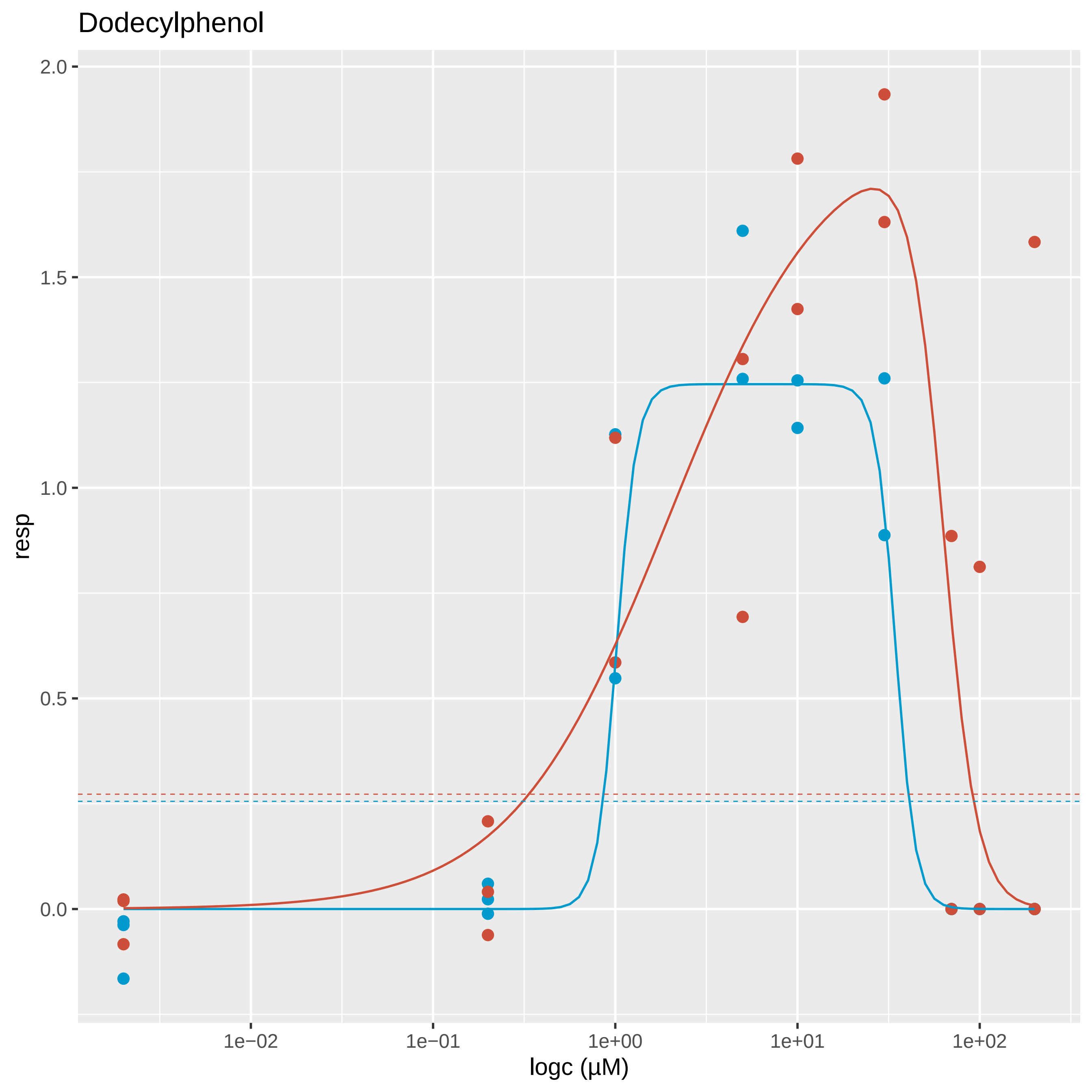
Dodecylphenol
resp
logc (µM)
2.0
1.5
1.0
0.5
0.0
1e−02
1e−01
1e+00
1e+01
1e+02

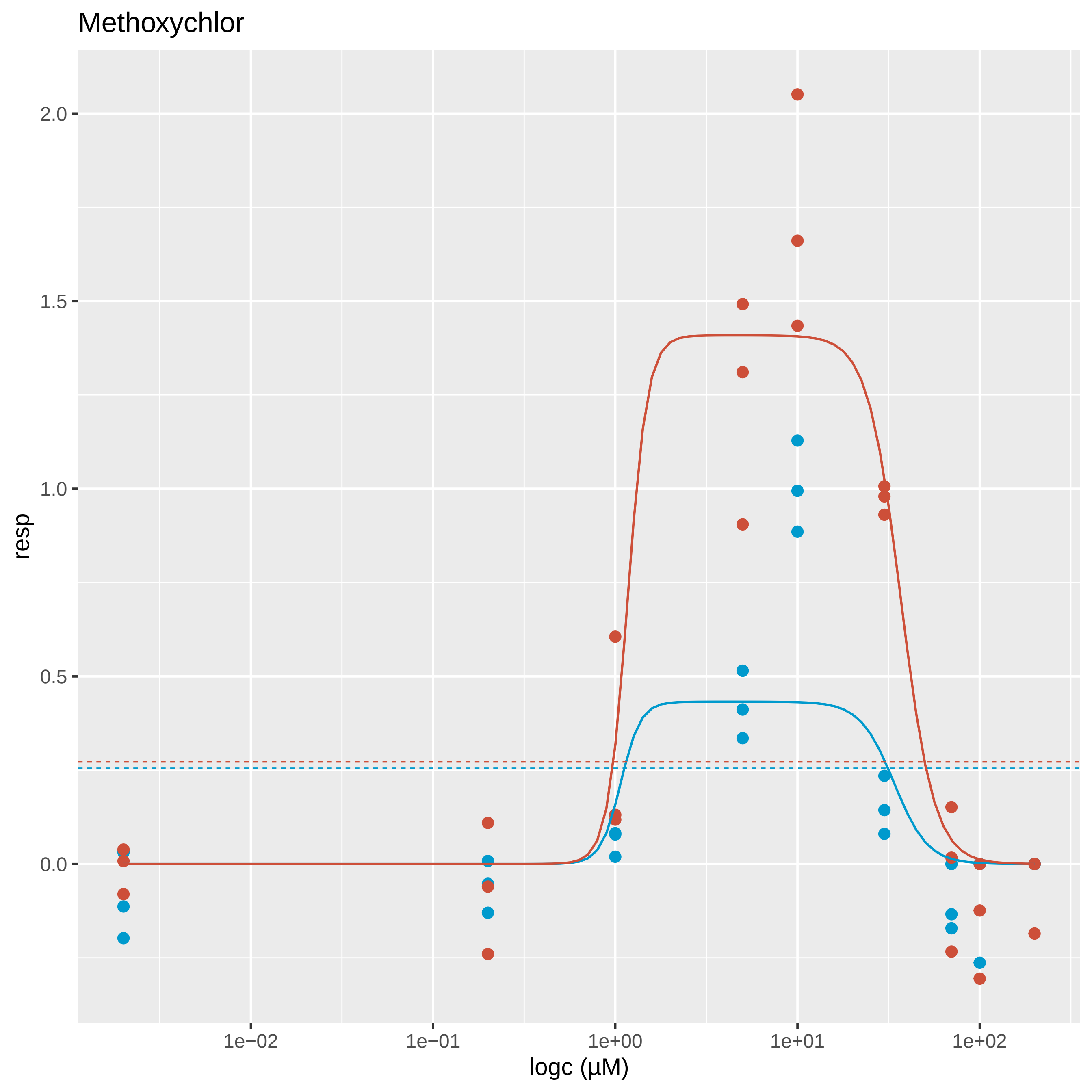

Methoxychlor
2.0
1.5
1.0
0.5
0.0
resp
1e−02
1e−01
1e+00
1e+01
1e+02
logc (μM)

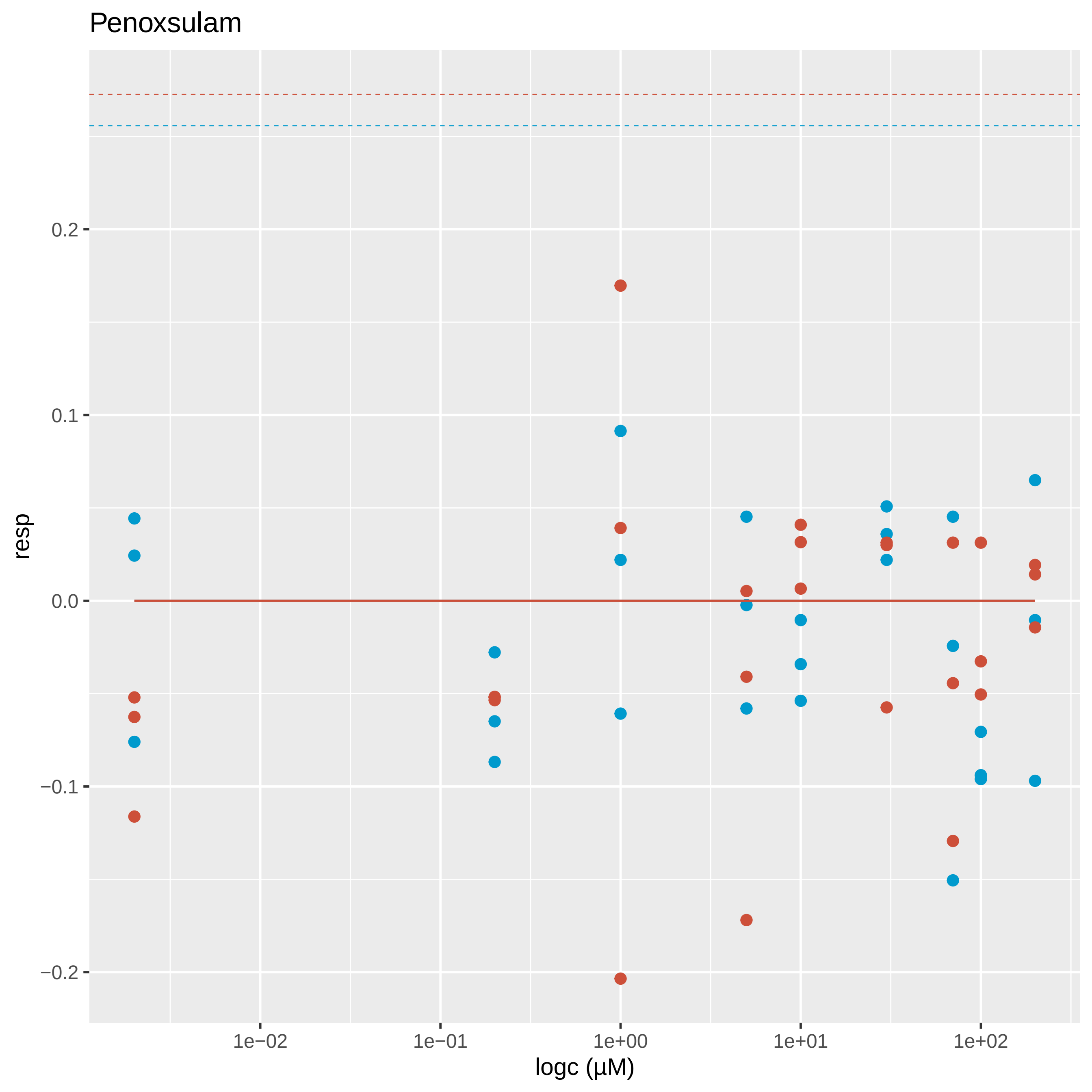

Penoxsulam
resp
0.2
0.1
0.0
−0.1
−0.2
1e−02
1e−01
1e+00
1e+01
1e+02
logc (μM)

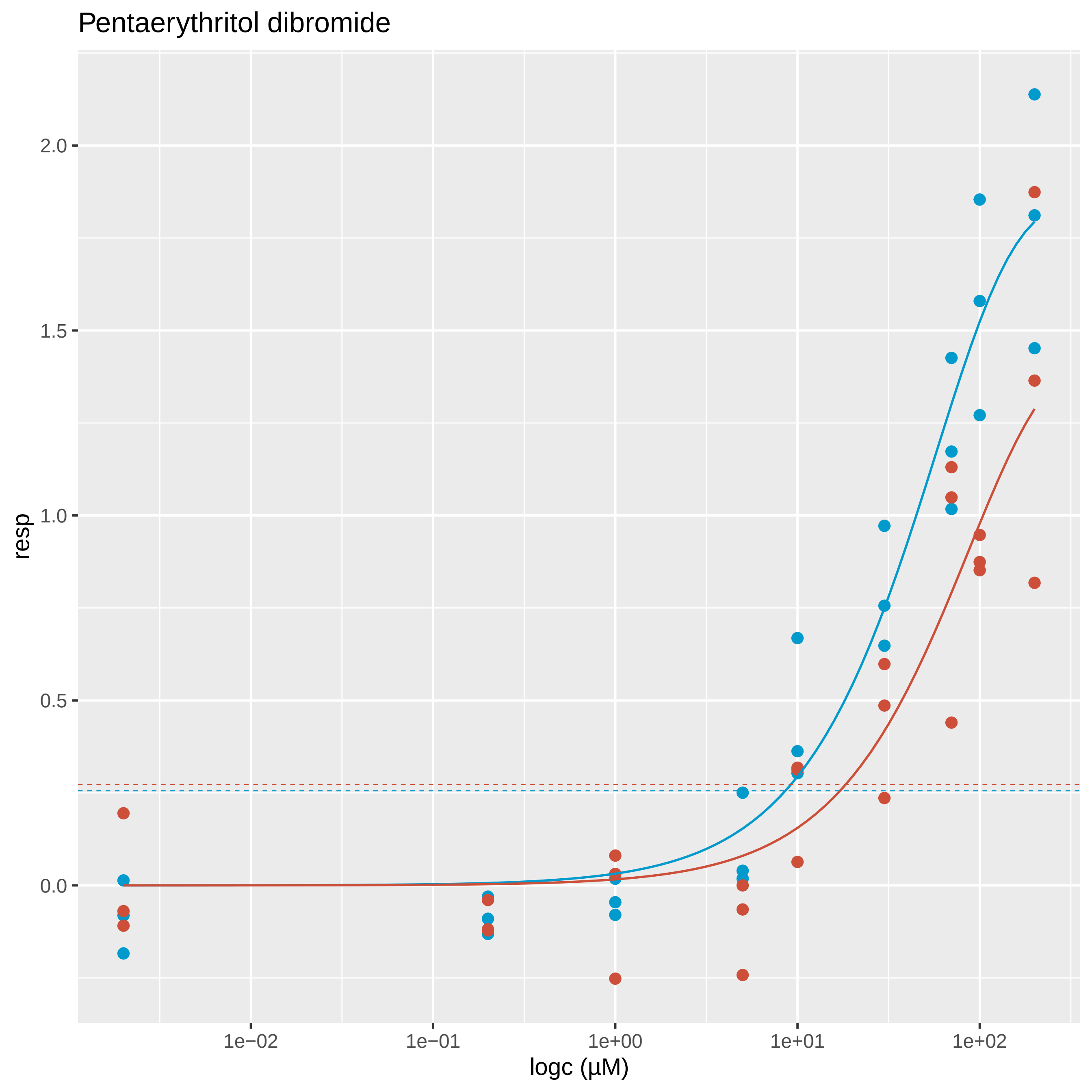

Pentaerythritol dibromide
resp
logc (µM)
2.0
1.5
1.0
0.5
0.0
1e−02
1e−01
1e+00
1e+01
1e+02

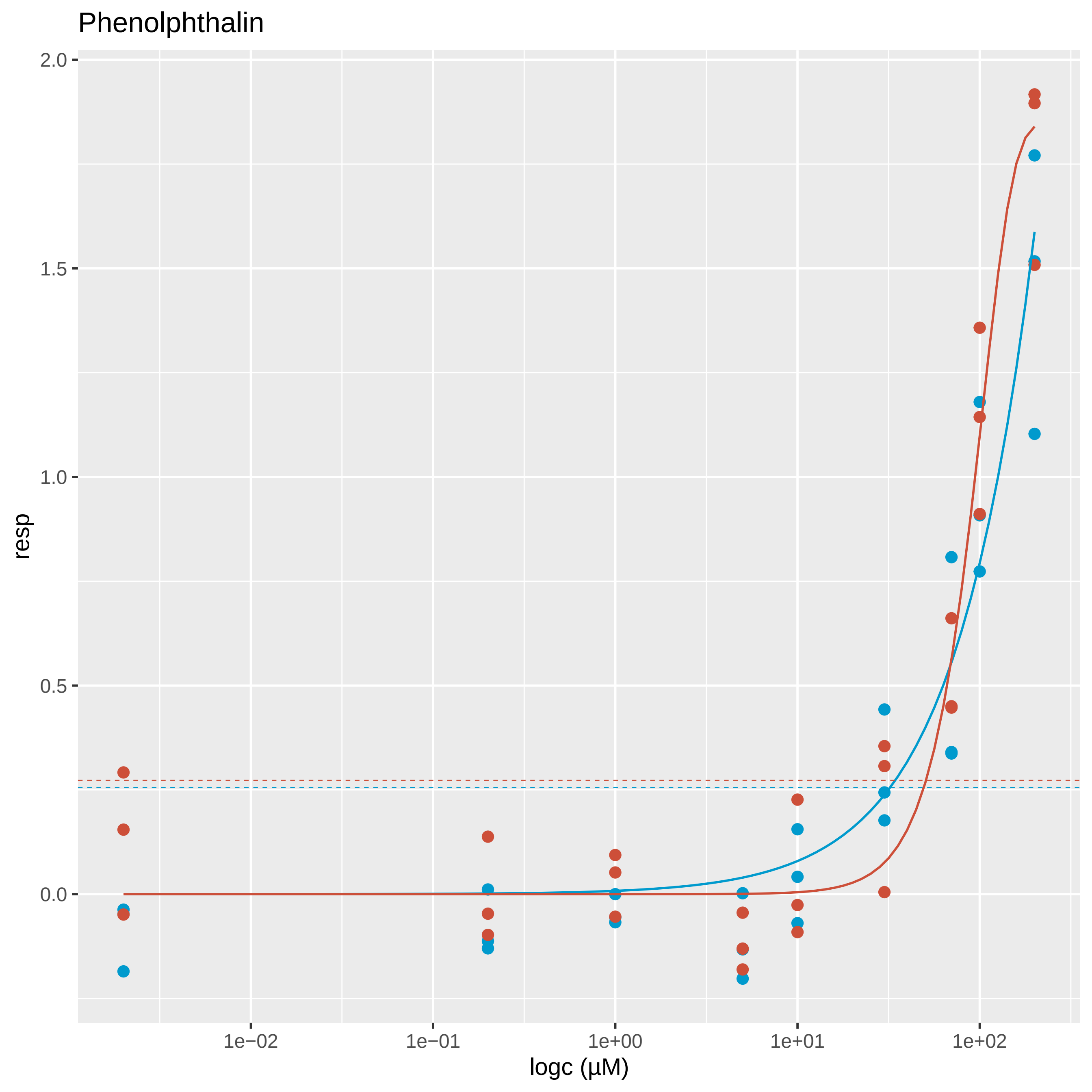

Phenolphthalin
resp
2.0
1.5
1.0
0.5
0.0
1e−02
1e−01
1e+00
1e+01
1e+02
logc (µM)

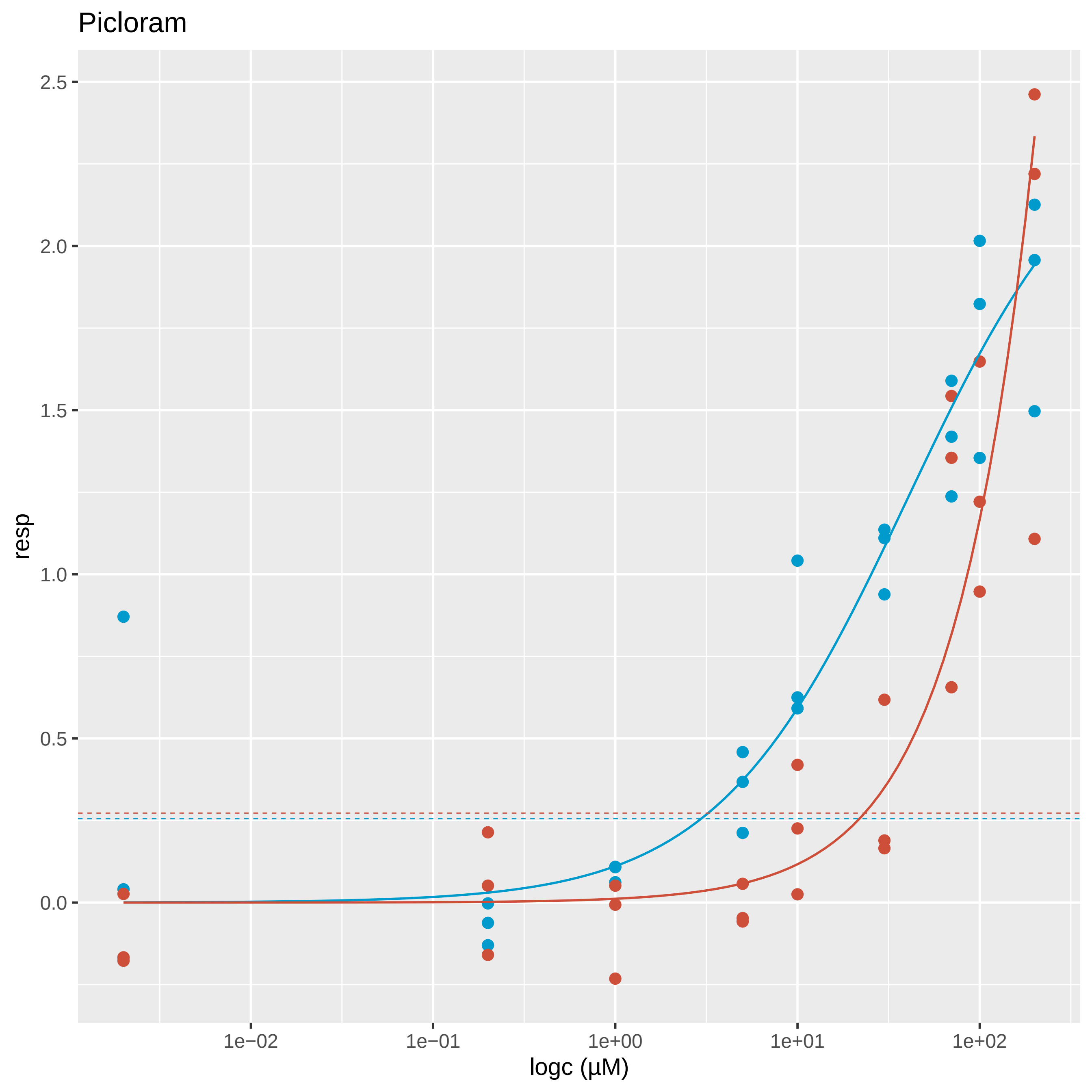

Picloram
resp
logc (µM)
2.5
2.0
1.5
1.0
0.5
0.0
1e−02
1e−01
1e+00
1e+01
1e+02

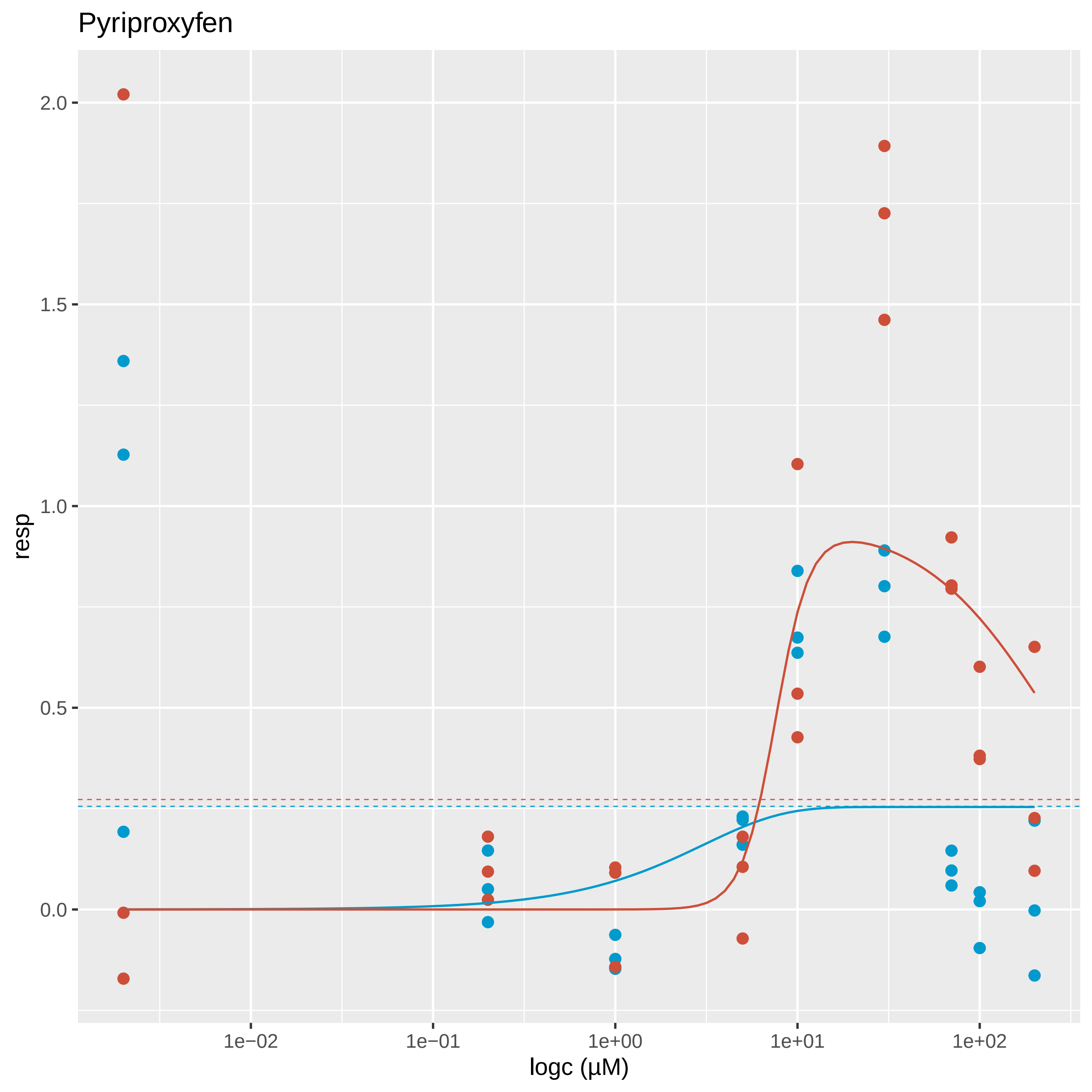

Pyriproxyfen
resp
2.0
1.5
1.0
0.5
0.0
1e−02
1e−01
1e+00
1e+01
1e+02
logc (µM)

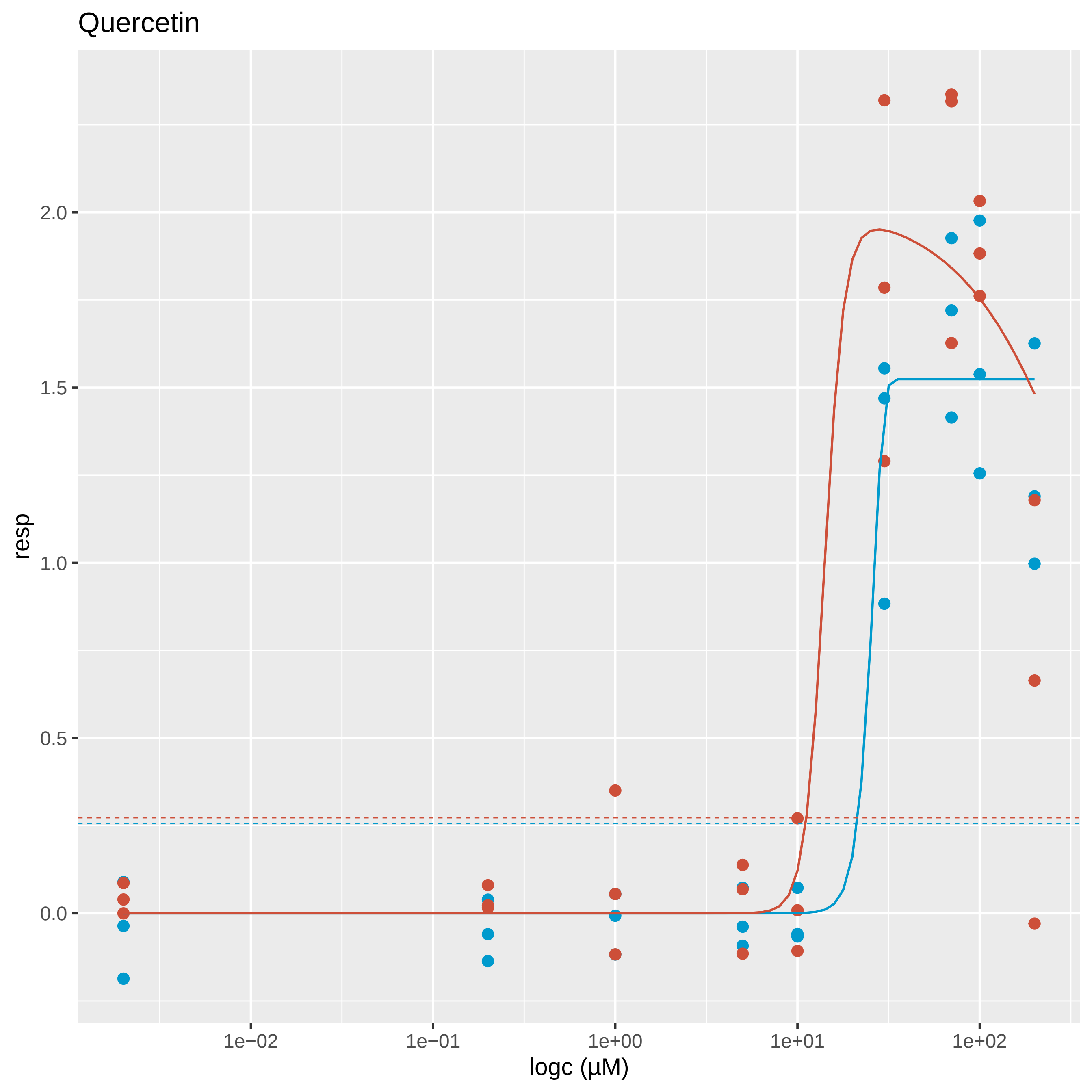

Quercetin
2.0
1.5
1.0
0.5
0.0
resp
1e−02
1e−01
1e+00
1e+01
1e+02
logc (µM)

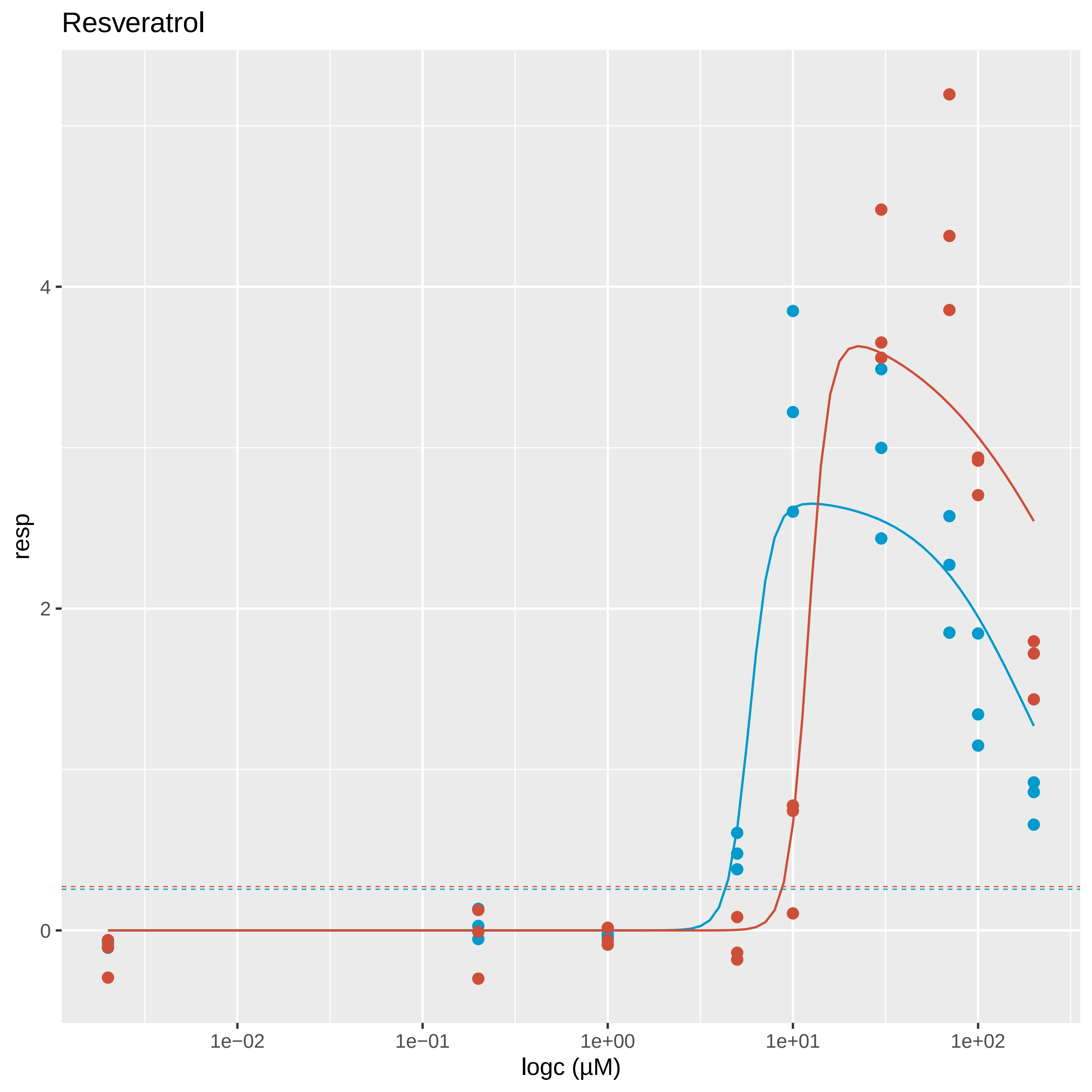

Resveratrol
resp
4
2
0
1e−02
1e−01
1e+00
1e+01
1e+02
logc (µM)

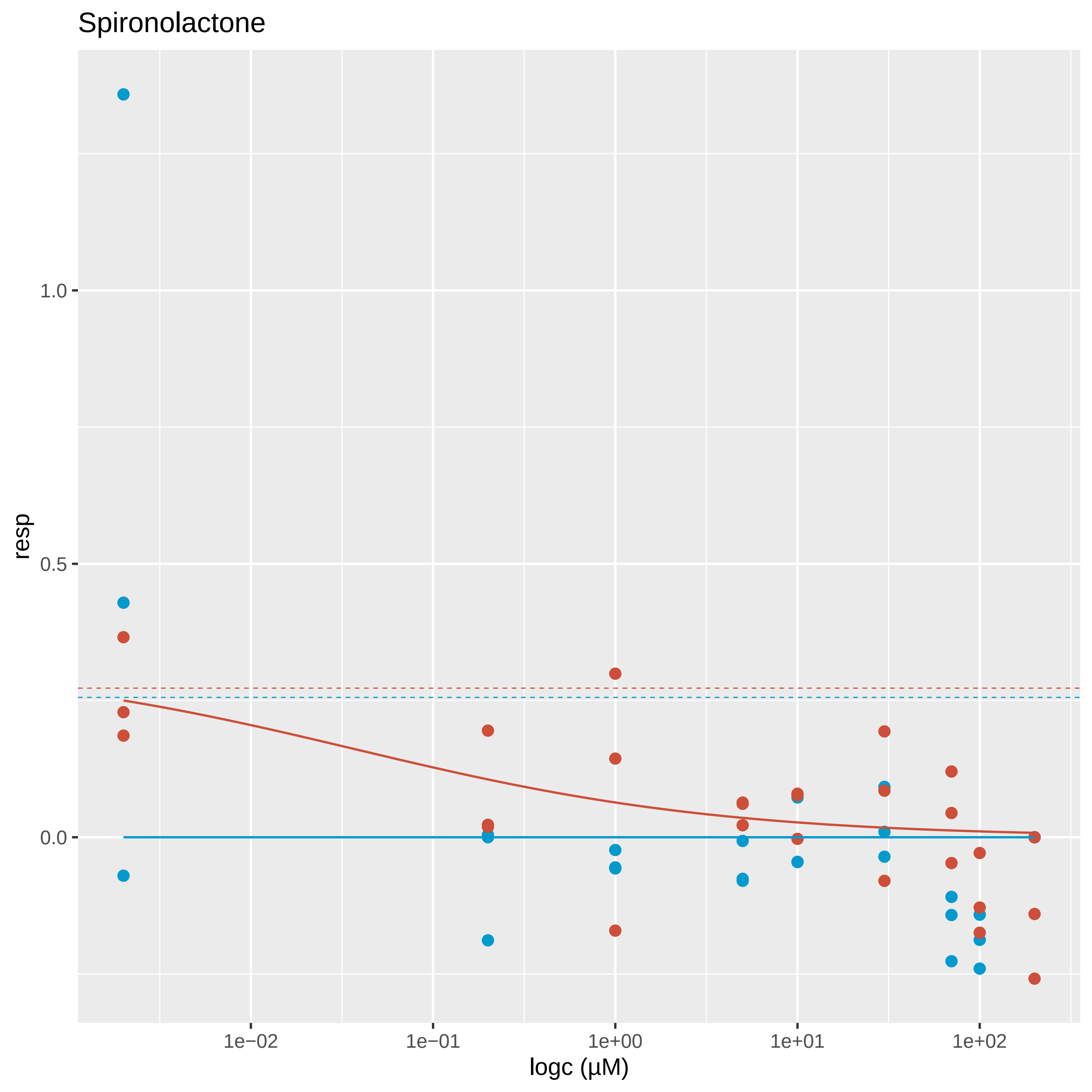

Spironolactone
1.0
0.5
0.0
resp
1e−02
1e−01
1e+00
1e+01
1e+02
logc (µM)
